# Mesenchymal stromal cells modulate infection and inflammation in the uterus and mammary gland

**DOI:** 10.1186/s12917-023-03616-1

**Published:** 2023-03-30

**Authors:** Iftach Schouten, Andrés Bernys-Karolys, Peleg Schneider, Tal Dror, Lior Ofer, Chen Shimoni, Einat Nissim-Eliraz, Nahum Y. Shpigel, Sharon Schlesinger

**Affiliations:** 1 Koret School of Veterinary Medicine, Faculty of Agriculture, Food and Environment, The Hebrew University of Jerusalem, POB 12, Rehovot, 76100 Israel; 2 Department of Animal Sciences, Faculty of Agriculture, Food and Environment, The Hebrew University of Jerusalem, POB 12, Rehovot, 76100 Israel

**Keywords:** Uterus, Metritis, Mastitis, Mesenchymal stromal cells, Murine model, *Escherichia coli*, Immunomodulation

## Abstract

The use of mesenchymal stromal cells (MSCs) is emerging as an efficacious and safe treatment for many infectious and non-infectious inflammatory diseases in human and veterinary medicine. Such use could be done to treat mastitis and metritis, which are the most common disease conditions affecting dairy cows leading to considerable economic losses and reduced animal welfare. Currently, both disease conditions are commonly treated using local and systemic administration of antibiotics. However, this strategy has many disadvantages including low cure rates and the public health hazards. Looking for alternative approaches, we investigated the properties of MSCs using in-vitro mammary and endometrial cell systems and in-vivo mastitis and metritis murine model systems. In-vitro, co-culture of mammary and uterus epithelial cells constructed with NF-kB reporter system, the master regulator of inflammation, demonstrated their anti-inflammatory effects in response to.LPS. In vivo, we challenge animals with field strains of mammary and utero pathogenic *Escherichia coli* and evaluated the effects of local and systemic application of MSC in the animal models. Disease outcome was evaluated using histological analysis, bacterial counts and gene expression of inflammatory markers. We show that MSC treatment reduced bacterial load in metritis and significantly modulated the inflammatory response of the uterus and mammary gland to bacterial infection. Most notably are the immune modulatory effects of remotely engrafted intravenous MSCs, which open new avenues to the development of MSC-based cell-free therapies.

**Supplementary Information:**

The online version contains supplementary material available at 10.1186/s12917-023-03616-1.

## Introduction

Metritis and mastitis, infection and inflammation of the mammary gland and uterus, respectively, are important disease conditions affecting dairy cows worldwide [1]. These disease conditions are leading causes of economic losses and reduced animal welfare [2]. Bacterial infection is the most frequent etiology of metritis and mastitis and antibiotics, which are commonly used for treatment and prevention, constitute a public health hazard to consumers of animal products. To this end, alternative therapeutic approaches are sought after and triggered our research studying the therapeutic use of mesenchymal stromal cells (MSC) for mastitis and metritis.

Mesenchymal stromal cells, also referred to as mesenchymal stem cells, identified as multipotent stromal cells, have multi-lineage differentiation ability and immunosuppressive properties [3]. They can be derived from several sources, including the umbilical cord, bone marrow, or fat tissue. We have recently reported the derivation and characterization of bovine MSC (bMSC) from umbilical tissues [4] and these cells were also used in this project.

Over the last 30 years, the medical utility of MSC-based therapy and its mode of action has been extensively studied. These efforts were recently analyzed and summarized in excellent review papers [5]. While animals (mostly rodents) were extensively used as translational in-vivo model systems [6], similar studies on farm animals are rare. This potential use of MSC in cattle was recently discussed [7]. Some in-vitro and in-vivo studies demonstrated encouraging immunomodulating, anti-inflammatory and antimicrobial effects in mammary and uterus cell systems and animal models [5, 8–13]. However, the feasibility and efficacy of MSC-based therapy for mastitis and metritis are still unknown and further research is required. To this end, we established in-vitro cell systems and in-vivo animal models, including a novel murine metritis model, which were used to study mouse and bovine MSC as potential therapeutics for mastitis and metritis.

## Materials and methods

### Mouse and bovine mesenchymal stromal cells

Mouse mesenchymal stromal cells (mMSC) were isolated, cultured and characterized as previously reported [14]. Cells were kindly provided by Dr. Osnat Almogi-Hazan, Hebrew University. Bovine mesenchymal stromal cells (bMSC), as we have previously reported [4], were isolated, cultured and characterized based on generally accepted criteria [3, 15]. For some experiments and microscopic imaging, cells were preincubated with carboxyfluorescein succinimidyl ester (CFSE; Celltrace, Invitrogen). Briefly, culture medium was removed and cells were treated with 10 μM CFSE in Dulbecco’s Phosphate Buffered Saline (DPBS, Biological Industries, Beit Haemek, Israel) and incubated for 20 min at 37°, 5% CO2. Cells were washed 3 times with a culture medium containing 10% FBS (Gibco), and incubated for further 10 min before harvesting or further processing.

### Bovine primary endometrial epithelial cells

Bovine primary endometrial cells were derived as previously described [16]. Briefly, whole uterus was obtained from a local abattoir and endometrial epithelium was dissociated using enzyme solution containing collagenase and deoxyribonuclease. Cells were filtered, tested for viability with trypan blue, enumerated and seeded into 48-well plates in DMEM/ Ham’s F-12 culture medium supplemented with 10% calf serum and antibiotics at 37^0^C in a humidified atmosphere of 5% CO_2_, 95% air. The cells were monitored daily and the media was changed 48 h after seeding to remove unattached cells until reaching confluence. After 6 days the cell medium was replaced with DMEM/Ham’s F-12 supplemented with 10% calf serum, 0.1% BSA, 5 ng/ml sodium selenite, 0.5 mM ascorbic acid, 5 mg/ml transferrin, and antibiotics.

### Bovine and mouse cell lines

Bovine Endometrial cells (BEND; ATCC CRL-2398) derived from the endometrium of a normal female cow on day 14 of the estrous cycle were cultured as previously reported [17]. Briefly, cells were cultured in a 1:1 mixture of DMEM and F12K medium supplemented with 10%FBS and 10% HS (D-Valine + , L-Valine +).

Mouse mammary epithelial line Eph4 and murine macrophage cell line RAW 264.7 were cultured as previously reported by us [18, 19].

### NF‑kB luminescence reporter system

Using lentivirus, primary endometrial cells, BEND cells, Eph4 cells and RAW 264.7 cells were transduced with luminescence NF-kB reporter system as previously reported by us [19, 20]. The luminescence reporter was constructed of destabilized firefly luciferase (pGL4.24-luc2P, Promega) open reading frame controlled by a DNA cassette containing five tandem repeats of the NF-kB transcriptional response element (pLNT-minP-5kB-luc2P). Following replacement of the luciferase gene by the venus gene (Yellow fluorescent protein), the same system was used to express fluorescence reporting system that enabled visualization of mMSCs in tissues (Supplementary Figure S6) [21, 22].

**Fig. 1:**
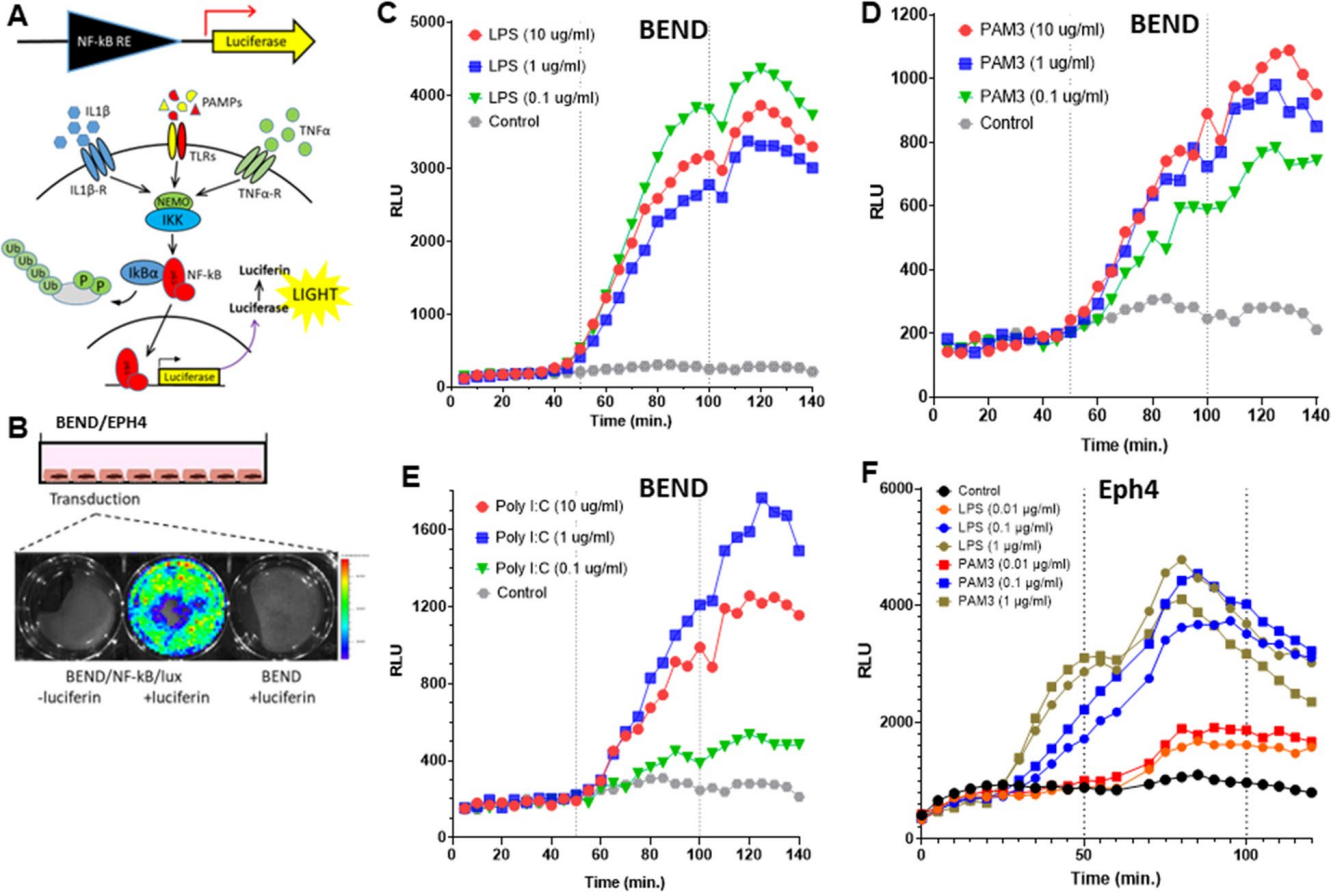
Activation of inflammatory response in endometrial and mammary epithelial cells. Bovine endometrial cell line (BEND) and mouse mammary alveolar epithelial cell line (Eph4) were transduced with luminescence NF-kB reporter (**A**-**B**). Activation of NF-kB, the master regulator of inflammation, in BEND cells is demonstrated in response to pathogen associated molecular patterns (PAMPs); LPS (**C**), PAM3CSK4 (**D**) and poly I:C (**E**). Activation of NF-kB in Eph4 cells in response to LPS and PAM3CSK4 is demonstrated (**F**). D-luciferin was added at time 0, 50 and 100 min (broken lines) and luminescence signals were quantified every 5 min using SpectraMax i3x multiple detection microplate reader (Molecular Devices, CA USA). Low resolution luminesce imaging of transduced BEND cells (**B**) was performed using IVIS Lumina Series III (PerkinElmer Inc., MA, USA)

All transduced cells were validated by luminesce and fluorescence signal imaging using IVIS Lumina Series III (PerkinElmer Inc., MA, USA) (see representative image in Fig. 1B) and luminescence signal quantification using SpectraMax i3x multiple detection microplate reader (Molecular Devices, CA, USA) in the presence of 150 µg/ml D-luciferin (GoldBio). LPS (from *E. coli* serotype O55:B5, Sigma, Rehovot, Israel), Pam3CSK4 (((S)-(2,3-bis(palmitoyloxy)-(2-RS)-propyl)-N-palmitoyl-(R)-Cys-(S)-Ser-(S)-Lys4-OH-3HCl, 06B24-MT; InvivoGen, CA, USA) or poly I:C (Poly(I:C) HMW; cat. # tlrl-pic-5, InvivoGen, CA, USA) were used for cell activation as specified in the result. The poly I:C water solution (1 mg/ml) was heated for 10 min at 67 °C and allowed to cool for 1 h at room temperature.

### Co-culture experiments

For co-culture experiments mouse or bovine MSCs were grown until reaching 80% confluence, thereafter cells were washed with DPBS and 0.25% trypsin–EDTA (Gibco) was applied to detach the cells. Cells were resuspended in culture medium, tested for viability with trypan blue and enumerated. BEND, PEP, Eph4 or RAW 264.7 transduced with the NF-kB luminescence reporter were seeded into 96-well plates and cultured in their respective media for 2–3 days to achieve confluent monolayers by the epithelial cells. MSCs were seeded on top of the co-cultured cells at ratios of 1:1 to 1:1000 MSCs to endometrial cells, mammary cells or macrophages and LPS (from *E. coli* serotype O55:B5, Sigma, Rehovot, Israel) was added at final concentration of 0.01–1 μgr/ml. Luminescence signals were quantified in the presence of 150 μg/mL D-luciferin (GoldBio) using SpectraMax i3x multiple detection microplate reader (Molecular Devices, CA, USA). Experiments were performed in triplicated replicates of all treatment conditions and each experiment was repeated at least 3 times.

Co-culture fluorescence imaging experiments were performed on cells grown on glass cover slides in 24 well culture plates. Eph4 cells were grown over 2–3 days to form a confluent monolayer and CFSE-stained MSCs were layered on top as described above. For fluorescence microscopy imaging cells were fixed with PFA and stained with DAPI (Sigma, Israel) and phalloidin-TRITC. Glass cover slides were mounted with VectaShield (Vector Laboratories, Burlingame, CA) and imaged with an Axio Imager M1 upright light microscope (Zeiss, Germany) coupled to a MR3 CCD camera system (ZEN 2012). Confocal images of Eph4-bMSC co-cultures were acquired with Leica TCS SP5 with a DN6000 microscope assisted by the LAS AF software (Leica). All images were processed with ImageJ (Wayne Rasband, NIH) using 3-D reconstructions and opacity mode.

### Murine mastitis model

Six- to eight-week-old, 7–10 days post-partum lactating BALB/c mice were used in this study (Envigo, Jerusalem, Israel). All mice were maintained under specific pathogen-free conditions and handled under protocols approved by the Hebrew University Animal Care Committee, according to international guidelines. IACUC approvals were obtained prospectively (Ethics Committee for Animal Experimentation, Hebrew University of Jerusalem; MD-18–15,523-3 and MD-18–15,453-3). Mice were challenged by intramammary (IMM) infusion through the teat canal as previously described, using a field strain of mammary pathogenic *E. coli* P4-NR [19] or 10 µgr LPS [23, 24].

MSC treatment was applied 6 h after challenge by either IMM infusion through the teat canal or intravenously through the tail vein of ~ 250,000 fluorescently-labeled (CFSE; Celltrace, Invitrogen) MSCs suspended in 50 µl PBS.

### Murine metritis model

Virgin, six- to eight-week-old female BALB/c mice were used in this study (Envigo, Israel). All mice were maintained under specific pathogen-free conditions and handled under protocols approved by the Hebrew University Animal Care Committee, according to international guidelines. Mice were assigned a treatment group at random. IACUC approvals were obtained prospectively (Ethics Committee for Animal Experimentation, Hebrew University of Jerusalem; MD-18–15,455-3 and MD-19–15,801-3).

Before challenge mice were treated with medroxy progesterone acetate (MPA; Depo-Provera, Pfizer Inc., New York City, NY, USA) for synchronization into diestrus stage. Mice were subcutaneously injected twice, on day 1 (starting day) and day 5, at the dose of 3 mg MPA per mouse and the synchronized diestrus stage was reached 7 days after first MPA administration [25]. The stage of synchronized mice was confirmed using vaginal smears [26] obtained before challenge on day 8. Ten minutes before challenge, mice were subcutaneously injected with 6 mg/kg clenbuterol hydrochloride (Planipart®, Boehringer Ingelheim, Auckland, NZ), a β2 receptor sympathomimetic to induces relaxation of the uterine musculature and dilation of the cervix. Transcervical intrauterine challenge was performed using *E. coli* strain 8-3B43 isolated from the uterus of 3 days post-partum commercial dairy cow clinically diagnosed with metritis as previously described [27, 28] (Kindly provided by Dr. Karen Wagener, University of Veterinary Medicine Vienna). Bacteria were transformed with plasmid pKB4985 which is based on pACYC184 constitutively expressing mCherry (low levels) [29]. Plasmid also include chloramphenicol resistance gene and to select for carriage of plasmid, growth medium was supplemented with chloramphenicol (10 µg/ml). Bacteria from frozen stocks were pre-cultured overnight on LB agar containing chloramphenicol, next, two colonies were picked and cultured in shaker incubator for 5 h at 37° C in LB broth with chloramphenicol (25 µgr/ml). Next, culture (~ 0.6 O.D.) was centrifuged for 10 min at 4000 rpm and bacteria were washed twice with sterile normal saline. Bacteria were resuspended and inoculum was prepared and quantified by plating serial tenfold dilutions on agar plates. For intra uterine challenge, mice were anesthetized (1.5—2.5% isofluorane in O_2_) and challenged by trans cervical infusion of 10 µl bacterial suspension using NSET™ Device (ParaTechs Corporation, Lexington, Kentucky, USA). Spillage of challenge was prevented by placing 30 µl of biological glue at the external os of the ectocervix. To enable intravital imaging of the challenged uterine horn, 5 µgr Indocyanine green (ICG) were added to the bacterial suspension and a whole-body image was acquired using IVIS Lumina Series III (PerkinElmer Inc., MA USA) with excitation/emission filters 780/845 for ICG fluorescence.

MSC treatment was applied 6 h after bacterial challenge using the procedures described above. Approximately 250,000 mMSC were suspended in 10 µl PBS, mixed with ICG and infused into the uterus. To enable visualization and tracking of mMSC following intrauterine treatment, lentivirus was used to transduce the cells with a fluorescence reporter as described above.

Control groups included diestrus (synchronized) normal, non-treated mice and diestrus mice infused with PBS or MSC as described above. Researchers were unblinded during sample collection in animals due to the importance of controlling for cross-contamination of bacteria across groups. Researchers were blinded to treatment groups when analyzing and quantifying samples.

### Histological analysis and bacterial counts

All mice were killed 24 h after challenge and mammary and uterus were trisected for histology, total RNA extraction and total bacterial counts as previously described [18, 19]. Mammary and uterus homogenates were plated as serial tenfold dilutions on LB agar plates with and without antibiotics and bacterial colonies were counted following incubation at 37 °C for 24 h to determine the output CFUs per 100 mg of tissue. Samples for histological analysis were fixed in neutral buffered 4% PFA and embedded in paraffin, and sections were cut at a thickness of 5 µm and stained with hematoxylin and eosin (H&E) according to standard procedures. Fresh mammary and uterus tissues for fluorescence staining was fixed in 4% PFA overnight at room temperature, incubated with 15% (wt/vol) sucrose for 72 h at 4 °C, and frozen in Tissue-Tek embedding medium (Electron Microscopy Sciences, Hatfield, PA). Serial 13-µm cryosections were stained with phalloidin (Sigma, Rehovot Israel) and DAPI (Sigma, Rehovot Israel). Immune cells were stained using anti-CD45 (ab23910), and goat anti-rat IgG (A11007) was used as the secondary antibody. Neutrophils were stained with rabbit anti-human Ly6G (EPR3094; Abcam) and donkey anti-rabbit IgG conjugated with Alexa Fluor 546 was used as secondary antibodies. ICAM-1 was stained using anti-CD54 conjugated antibody IgG2b (Biolegend). Sections were mounted with Vectashield mounting medium (Vector Laboratories, Burlingame, CA) and imaged with an Axio Imager M1 upright fluorescence microscope (Zeiss, Germany). Histological analysis was carried out in a blinded manner on H&E stained uterus samples from experimental groups and the levels of disease were scored as described in Supplementary Table 1 which was adopted from previous publication [30].

### RT-qPCR

Quantitative PCR (qPCR) was performed as previously described [19, 31]. Total RNA was isolated from mammary and uterus tissues using the GeneElute Mammalian Total RNA Miniprep Kit (Sigma, Rehovot, Israel) combined with on-Column DNase I Digestion Set (Sigma, Rehovot, Israel).

Reverse transcription was performed using qScript cDNA Synthesis Kit (Quanta BioSciences, Gaithersburg, MD, USA) and cDNA was used for subsequent qPCR reactions. Quantitative RT–PCR was conducted on a StepOne Plus PCR instrument (Applied Biosystems) using the FAST qPCR Universal Master Mix (Kappa Biosystems, Boston, MA, USA). PCR primers used in this study are listed in Supplementary Table 2. All reactions were performed in triplicates and the gene expression levels for each amplicon were calculated using the ΔΔCT method and normalized against HSP90 mRNA.

### Intramammary application of bMSC in lactating dairy cows

Three normal lactating dairy cows in the Volcani Dairy Farm (ARO, Israel ministry of agriculture) were selected based on prior milk somatic cell counts (SCC < 100,00/ml) and negative bacterial cultures. All milk SCC and bacteriological cultures were performed by the National Service for Udder Health and Milk Quality (NSUHMQ) laboratory. Quarter milk samples were obtained and submitted to NSUHMQ laboratory and SCC and bacteriological examination were performed according to National Mastitis Council guidelines [32].

Two diagonal quarters were infused with 10^6^ bMSC labelled with a fluorescent dye (CFSE; Celltrace, Invitrogen) in 10 ml normal saline. The contralateral quarters of each cow were infused with 10 ml normal saline as controls. Milk production, rectal temperature, blood total white cell count, milk SCC, and clinical mastitis score (CMS; 7–35-point scale) [33] were recorded daily before MSC infusion and 8 hourly thereafter. Cows were humanely sacrificed 24 h after bMSC treatment and supramammary and prescapular lymph nodes and udder tissues from all quarters were taken for histological and RT-qPCR analyses.

Cows were maintained and handled under protocols approved by ARO Animal Care Committee and Use Committee and approvals were obtained prospectively (Ethics Committee for Animal Experimentation, Agriculture Research Organization, Ministry of Agriculture and Rural Development; #11,380).

### Quantification and statistical analysis

All experiments were performed at least three times and representative images were chosen for publication. Where appropriate, the number of experiments is given (individual mice or biological replicates if not stated otherwise). The relative levels of expression of genes, metritis score and CFU counts were calculated as the median and for comparison between groups, a nonparametric Mann–Whitney two independent-samples test was applied using GraphPad Prism (version 6) software (Graph-Pad Software, Inc.). *P* values less than 0.05 were considered significant and presented as ∗ when *p* < 0.05; ∗  ∗ when *p* < 0.01; ∗  ∗  ∗ when *p* < 0.001. No significance was presented as ns.

## Results

### MSC modulate NF-kB response in cultured cells

Mammary alveolar and endometrial epithelial cells constitute the first line of response to luminal microorganisms following ascending infections of the mammary and uterus, respectively. Noteworthy, the role of endometrial macrophages was highlighted by previous studies [34, 35] and we have previously demonstrated that mammary alveolar macrophages are also essential in the initial response to luminal LPS [24]. To study the effects of MSCs on the inflammatory response activated by essential microbial components, we established in-vitro epithelial and macrophage cell systems. Using luminesce NF-kB reporter system (illustrated in Fig. 1A-B) we first demonstrated activation of this master regulator of inflammatory response [36] with LPS (TLR4 agonist; Fig. 1C), PAM3CSK4 (TLR2 agonist; Fig. 1D) and poly I:C (TLR3 agonist; Fig. 1E) in bovine endometrial cells (BEND) and murine mammary alveolar epithelial cells (Eph4) (Fig. 1F). Similar validation performed in macrophage cell lines was previously reported by us [21].

**Fig. 2:**
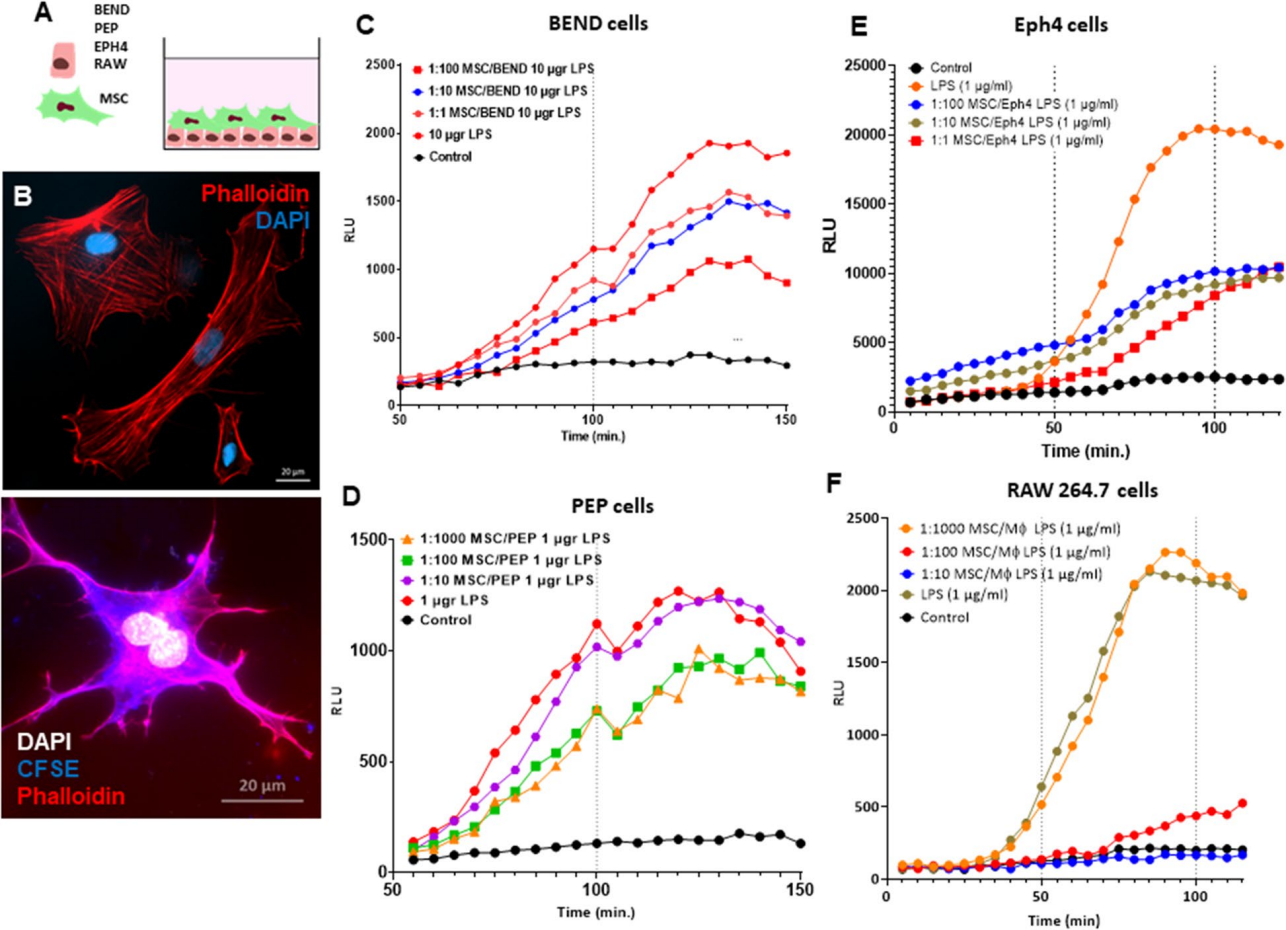
Bovine mesenchymal stromal cells (bMSC) suppress activation of NF-kB by LPS in endometrial cells, mammary epithelial cells and macrophages. Bovine endometrial cells (BEND), bovine primary endometrial epithelial cells (PEP), mouse mammary epithelial cell line (Eph4) or mouse macrophages cell line (RAW 264.7) expressing the luminescence NF-kB reporter were co-cultured with bMSC and activated with LPS (**A**). Fluorescence microscopic images of bMSC stained with DAPI, phalloidin and CFSE are shown in (**B**). Co-culture experiments were performed at 1:1, 1:10, 1:100 and 1:1000 ratios of bMSC to BEND (**C**), PEP (**D**), Eph4 (**E**) or RAW 264.7 (**F**) cells. D-luciferin was added at time 0, 50 and 100 min (broken lines) and luminescence signals were quantified every 5 min using SpectraMax i3x multiple detection microplate reader (Molecular Devices, CA USA). Scale bars 20 µm

Next, we co-cultured increasing quantities of bMSC with these epithelial cells and RAW 264.7 macrophages (as illustrated in Fig. 2A) to analyze the effects of bMSCs (Fig. 2B) on NFkB activation in these cell systems. We show here that in normal steady state conditions bMSC did not activate inflammatory response in bovine endometrial cells (Fig. 2C and D), mammary epithelial cells (Fig. 2E), or RAW macrophages (Fig. 2F) while suppressing NF-kB activation in response to LPS. Moreover, a low ratio of MSC to epithelial cells or macrophages in the co-culture was sufficient to considerably suppress the activation triggered by LPS.

To better understand the interactions between the co-cultured cells we performed fluorescence microscopic imaging and analysis of co-cultured bMSC and Eph4 cells. Surprisingly, we show here the emergence of the complex spatial organization formed by dispersed MSCs organized into fractal-like clusters on top of the epithelial monolayer (Supplementary Figure S1). These clusters exhibited a branched architecture and confocal microscopic analysis revealed a continuous network of interconnected MSCs.

Following our in-vitro results our next aims were to study the effects of MSCs on steady state and inflamed mammary gland and uterus. To this end, we developed a novel murine metritis model in virgin mice and we have used the murine mastitis model system in lactating mice.

### Establishment of murine metritis model

**Fig. 3:**
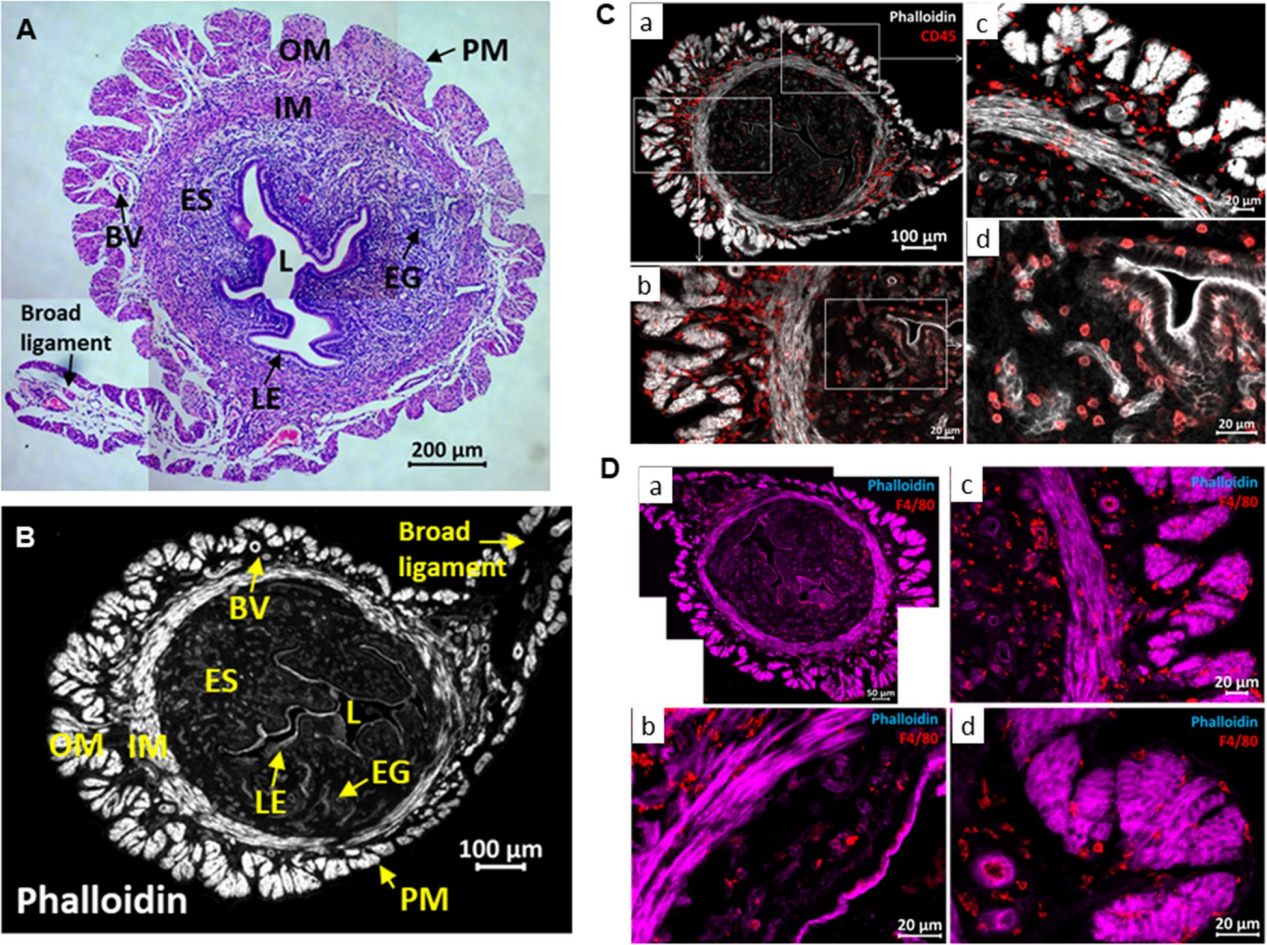
The normal diestrus murine uterus. Synchronized diestrus virgin 6–8 weeks old female BALB/C mice were used as normal controls to establish the structure, immune cells composition and expression of inflammatory markers in steady state uteri. Annotated microscopic images of H&E stained FFPE section (**A**) and cryo sections stained with Phalloidin (**B**-**D**). Annotated structural elements include; lumen (L), luminal epithelium (LE), endometrial glands (EG), endometrial stroma (ES), inner muscles (IM), outer muscles (OM), blood vessels (BV), perimetrium (PM) and broad ligament. Immune fluorescence staining was used to demonstrate CD45 + immune cells in the normal diestrus uterus (**C**a-d), most of which were F4/80 + macrophages (**D**a-d). Staining intensities were subjected to nonlinear adjustments so that structures would be visible at low magnification. Scale bars; 200 µm (**A**), 100 µm (**B**-**C**a), 50 µm (**D**a) and 20 µm (**C**b-d, **D**b-d)

**Fig. 4:**
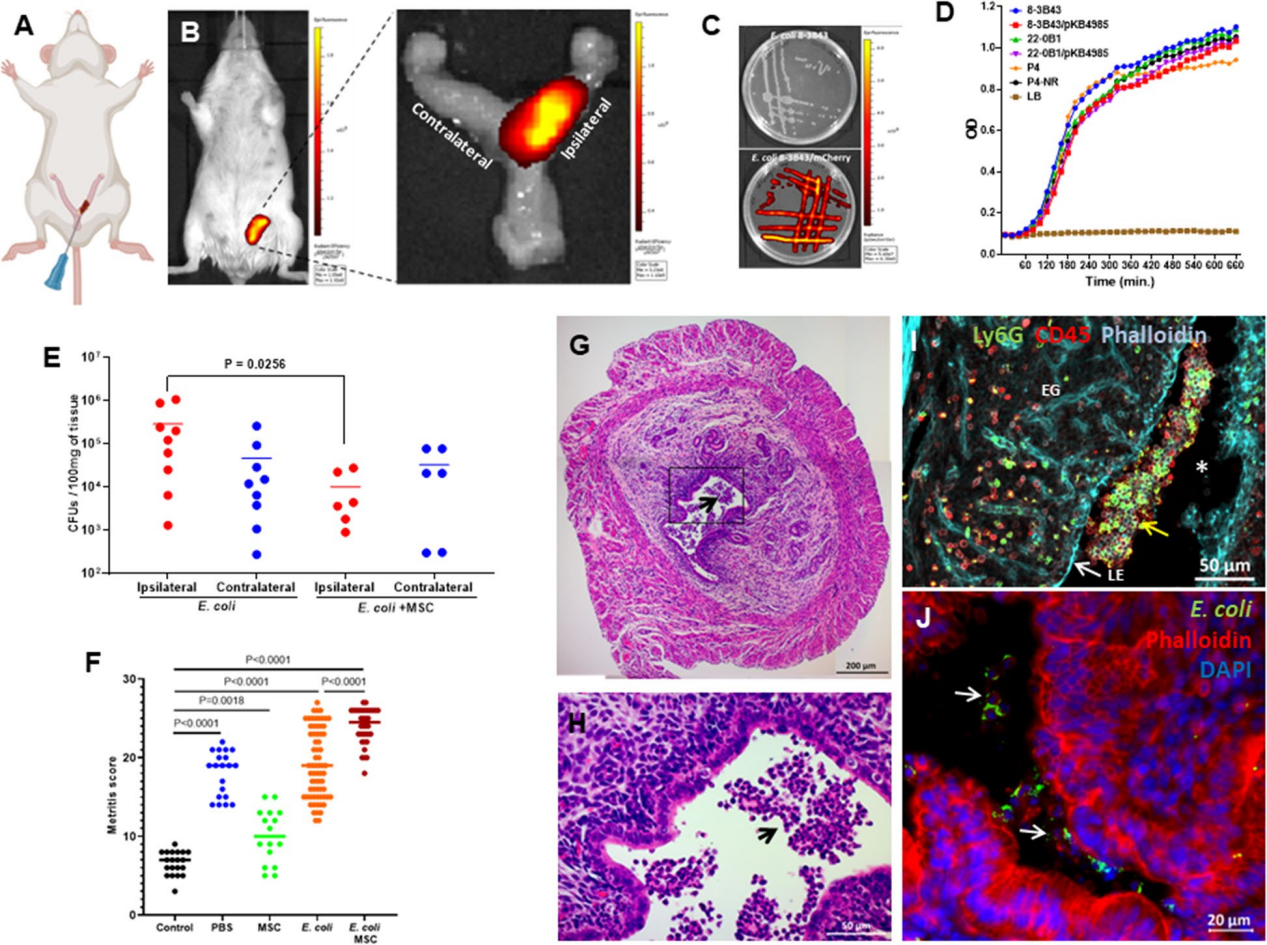
Establishment of the murine metritis model system and mMSC therapy. Imaging of intrauterine transcervical infusion (**A**-**B**). *E. coli* strain 8-3B43/pKB4985 mCherry expressing bacteria originally isolated from a natural case of clinical post-partum bovine metritis in a dairy cow (**C**). Comparative growth curves in LB of utero pathogenic (8-3B43 and 22-0B1) and mammary pathogenic (P4 and P4-NR) *E. coli* strains (**D**). Bacterial colonization observed following *E. coli* challenge was significantly reduced by MSC treatment of challenged mice (**E**). Metritis was scored (**F**) using H&E stained sections (**G**-**J**). Metritis is characterized by massive infiltration of neutrophils into the endometrium and lumen (black arrows in **G**-**H** and yellow arrow in **I**). Representative images of fluorescence staining using DAPI (blue) phalloidin (cyan in **H** and red in **I**), anti-murine CD45 antibodies (red in **I**), anti-murine Ly6G (green in **I**). Fluorescing mCherry bacteria interacting with luminal neutrophils and endometrial epithelial cells are visible in (**J**). Some challenged mice were also treated with mMSCs and disease was evaluated by metritis score (**F**). In scatter plots (**E** and **F**), each data point represents a single uterine horn and each experimental group was compared to the normal control group. Statistical significance was determined by non-parametric Mann–Whitney two-independent-samples test using GraphPad Prism 6 (GraphPad Software, Inc.) and *P* value of 0.05 or less was considered significant. Scale bars; 200 µm (**G**), 50 µm (**H**-**I**) and 20 µm (**J**). Figure (**A**) was created with BioRender.com

**Fig. 5:**
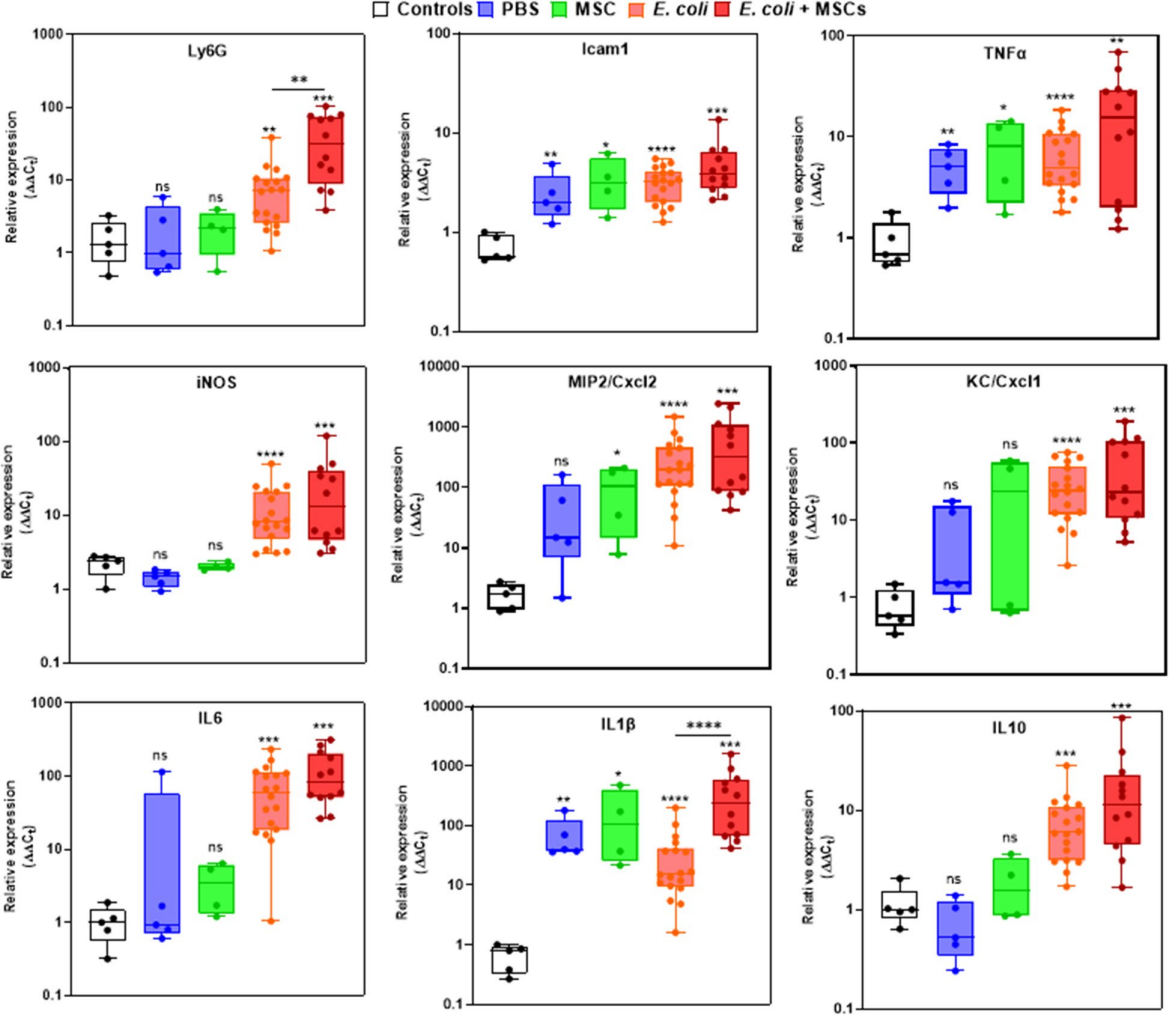
Relative expression of inflammatory markers in the uterus. Six to eight weeks old diestrus virgin BALB/C mice were treated by transcervical infusion with (1) PBS, (2) mMSC, (3) *E. coli*, or (4) *E. coli* followed by mMSC six hours thereafter. Normal, non-treated diestrus mice were used as controls. Mice were sacrificed 24 h after challenge and total RNA was extracted from uterine horns. Using RT-qPCR the relative expression of Ly6G (neutrophil marker), ICAM1, TNFɑ, iNOS, MIP2, KC, IL6, IL1β and IL10 genes was quantified relative to RNA samples extracted from the uterus of normal age-matched, diestrus control mouse. Each data point within box plots represents a single uterine horn and data demonstrates recruitment of neutrophils (Ly6G) and increased expression of inflammatory markers in the uterus. Each experimental group was compared to the normal control group or between groups and statistical significance was determined by non-parametric Mann–Whitney two-independent-samples test using GraphPad Prism 6 (GraphPad Software, Inc.) and *P* value of 0.05 or less was considered significant. Non-significant; (ns); *P* > 0.05, *P ≤ 0.05; **P ≤ 0.01, ***P ≤ 0.001, ****P ≤ 0.0001

We show here the establishment of a reliable murine metritis model system. We first established fluorescence microscopic techniques and gene expression assays that enabled us to analyze structural elements, immune cells and inflammatory markers in the normal steady state diestrus uterus before and following infection. The diestrus normal uterus (Fig. 3A-B) was richly populated by CD45^+^ immune cells (Fig. 3C), most of which localized in the muscular layers and around the vascular network system between the longitudinal and circular muscular layers and the periuterine lymphatics. Using immunohistochemical staining we further characterized most of these resident immune cells as F4/80^+^ macrophages (Fig. 3D) while neutrophils were not visible. Next we established transcervical catheterization techniques (Fig. 4A-B) that enabled uterine challenge of diestrus virgin BALB/c mice with *E. coli* 8-3B43/mCherry (Fig. 4C-D), a strain previously isolated from a field case of bovine metritis. Infection (see bacterial counts in Fig. 4E) and inflammation (see metritis score in Fig. 4F) of the uterus were characterized by massive recruitment of blood neutrophils into the endometrium and uterine lumen demonstrated using microscopy (Fig. 4G-J and Supplementary Figure S2-S3) and further supported by elevated expression of the neutrophil marker Ly6G (Fig. 5; top left panel). Although the endometrium was highly populated with F4/80^+^ macrophages these cells were not visible in the lumen of the infected uterus which was strictly populated by neutrophils (Supplementary Figure S4). Gene expression of other inflammation markers; Icam1 (ICAM-1), Tnf (TNFɑ), Nos2 (iNOS), Cxcl2 (MIP2), Cxcl1 (KC), Il6 (IL-6), Il1b (IL-1β) and Il10 (IL-10), was also significantly elevated (Fig. 5).

Elevated Icam1 gene expression was further validated and spatialy resolved using immunofluorescence microscopy. We show here that in murine metritis, the expression of ICAM-1 was dramatically increased in vascular endothelial cells and endometrial epithelial cells of the inflamed uterus (Supplementary Figure S5) suggesting that ICAM-1 is an important component of the molecular mechanism enabling recruitment of blood neutrophils in response to luminal bacterial infection.

### Effects of MSC in murine metritis model

We next analyzed the effects of transcervical application of mMSCs into the uterine lumen of normal and metritis diestrus virgin mice. Six to eight weeks old diestrus virgin BALB/C mice were allocated to the following treatment groups; (1) PBS, (2) mMSC-Venus, (3) *E. coli* 8-3B43/mCherry, and (4) *E. coli* 8-3B43/mCherry followed by mMSC-Venus six hours thereafter.

**Fig. 6:**
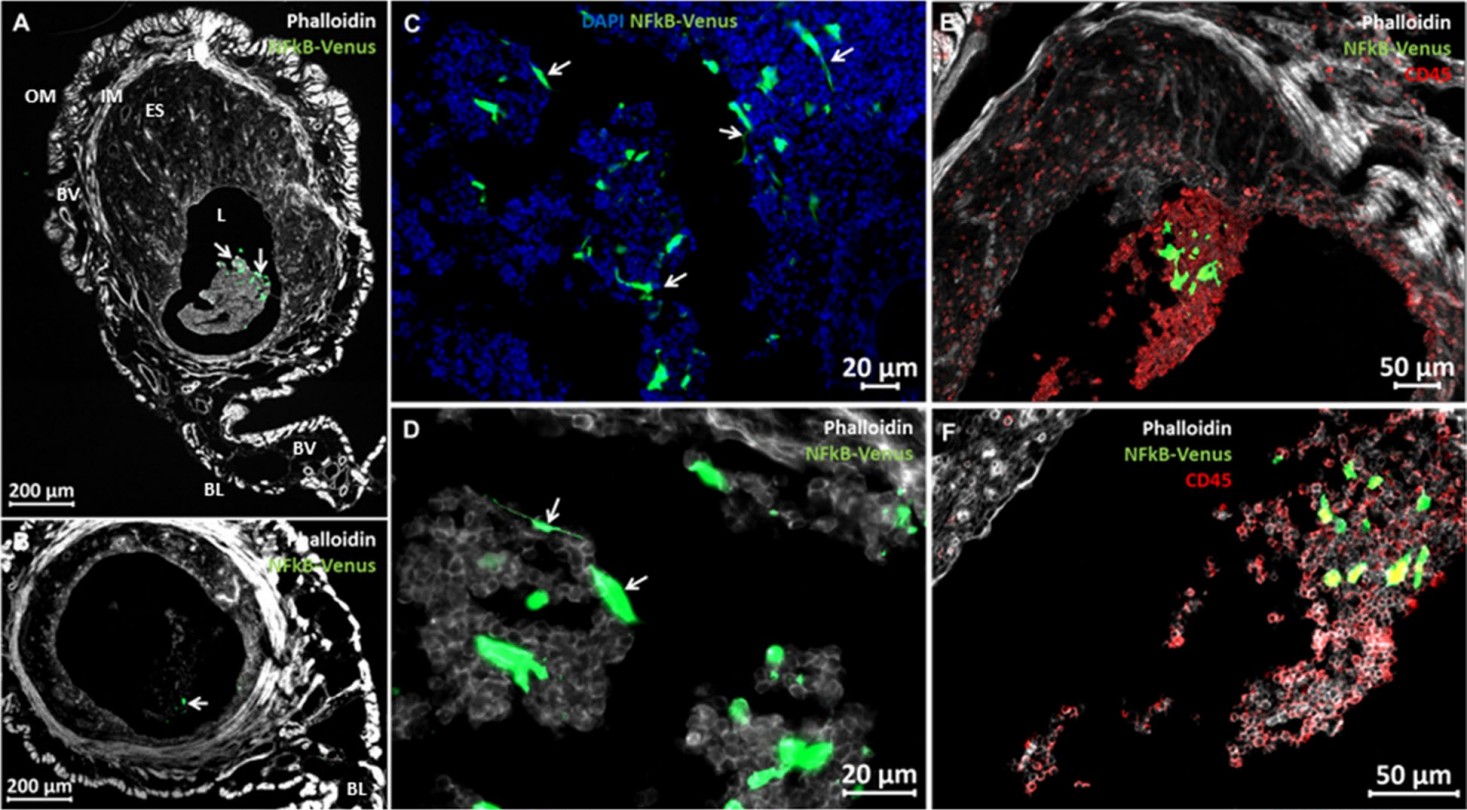
Luminally infused murine mesenchymal stromal cells (mMSC) interacting with neutrophils. Diestrus virgin mice were challenged with *E. coli* bacteria and treated 6 h later with mMSC transduced with the NF-kB/venus reporter system. Both challenge and treatment were applied by transcervical infusion. Mice were sacrificed 24 h after challenge and uterus tissue sections were prepared from PFA-fixed cryo blocks. mMSC (green) are visible in both ipsilateral and contralateral horns (white arrows in **A** and **B**, respectively). Representative images of fluorescence staining using phalloidin (white), anti-murine CD45 antibodies (red in **E**–**F**) and DAPI (blue in **C**). Annotated structural elements include; lumen (L), endometrial stroma (ES), blood vessels (BV), inner muscles (IM), outer muscles (OM) and broad ligament (BL). mMSC assume variable morphologies from spindle-shaped to large globular (**C**-**D**) interacting with luminal neutrophils (CD45^+^ cells in **E**–**F**). Scale bars; 200 µm (**A**-**B**), 20 µm (**C**-**D**), 50 µm (**E**–**F**)

Animals were sacrificed 24 h after challenge and uterus tissue samples were cultured for bacterial growth and processed for microscopic and RT-qPCR analysis. Bacterial colonization observed following *E. coli* challenge was significantly reduced by MSC treatment of challenged mice (Fig. 4E) while metritis score (Fig. 4F) and immune response were significantly higher (Fig. 5). Fluorscent mMSC (Supplementary Figure S6) were clearly visible in the uterine lumen interacting with luminal neutrophils (Fig. 6). However, we could not demonstrate mMSC in the endometrial tissue beyond the endometrial epithelial layer even though this barrier was compromized in many of the samples.

Interestingly, transcervical intrauterine infusion of similar voulmes of PBS or MSCs suspended in PBS also elicited an inflammatory response albeit somewhat different (Fig. 5). Most notably, the expression of Ly6G (Fig. 5; top left panel), iNOS (Fig. 5; middle left panel) and IL-10 remained basal (Fig. 5; bottom right panel) while significantly elevated following bacterial challenge.

### Effects of MSC on murine mammary glands

We first tested the effect of intramammary (IMM) treatment with bMSCs on the lactating murine.

**Fig. 7:**
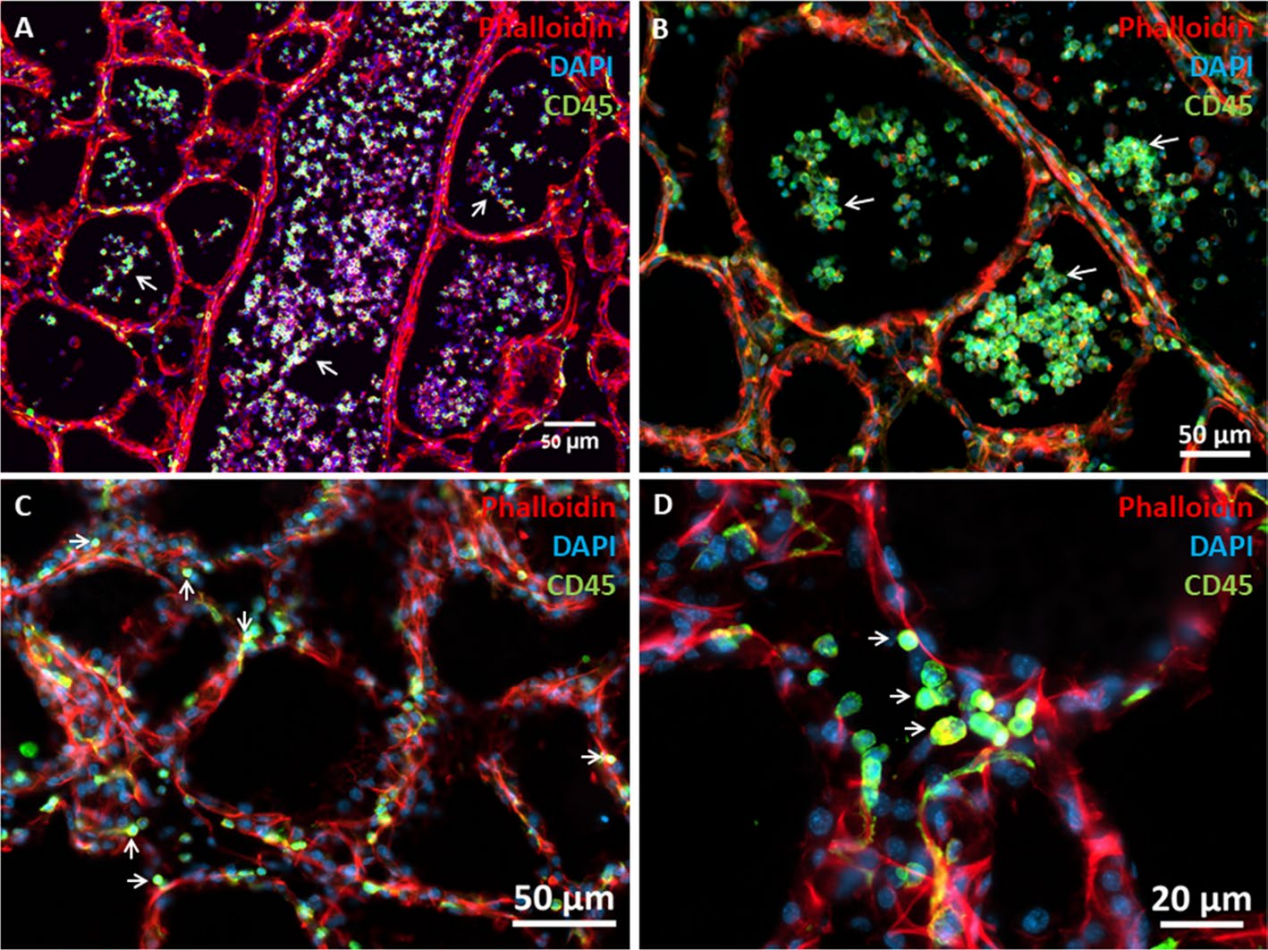
Bovine MSC elicits inflammatory response in the mouse mammary gland. Lactating mice were challenged by intramammary (IMM) infusion of LPS (**A**-**B**) or bMSC (**C**-**D**). Representative fluorescent microscopy images of mammary tissues 24 h after IMM infusion stained with DAPI (blue), phalloidin (red) and anti mouse CD45 antibodies (green). Inflammatory response in LPS-treated glands is characterized by massive recruitment of blood neutrophils into alveoli and milk ducts (white arrows in **A**-**B**), while neutrophil recruitment after bMSC treatment is restricted to the parenchyma surrounding the alveoli (white arrows in **C**-**D**) and were not visible in the alvolar milk space. Scale bars; 50 µm (**A**-**C**), 20 µm (**D**)

**Fig. 8:**
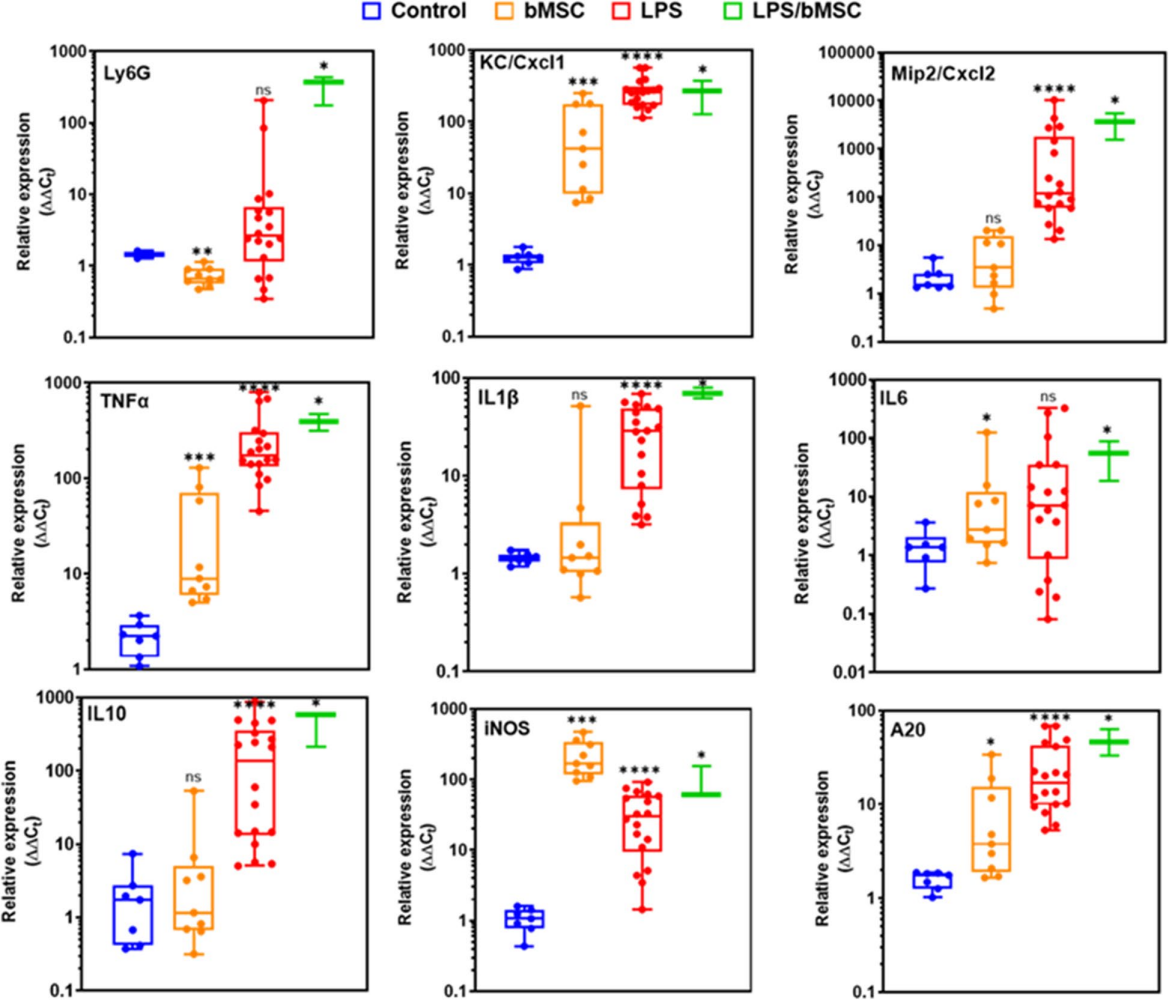
Relative expression of inflammatory markers in mammary glands. Normal lactating mammary glands (Control; blue), and 24 h after challenge with bMSC (orange), LPS (red) or LPS followed by bMSCs six hours thereafter (green) the relative expression of Ly6G (neutrophil marker), KC/Cxcl1, Mip2/Cxcl2, TNFɑ, IL1β, IL6, IL10, iNOS, and A20 genes was quantified. Each data point within box plots represents a single gland and data demonstrates recruitment of neutrophils (Ly6G) and increased expression of inflammatory markers. Each experimental group was compared to the normal control group or between groups and statistical significance was determined by non-parametric Mann–Whitney two-independent-samples test using GraphPad Prism 6 (GraphPad Software, Inc.) and *P* value of 0.05 or less was considered significant. Non-significant; (ns); *P* > 0.05, *P ≤ 0.05; **P ≤ 0.01, ***P ≤ 0.001, ****P ≤ 0.0001

Bovine MSCs were suspended in PBS (100,000 cells in 50 µl) and infused through the teat canal into the mammary glands of lacating BALB/c mice 7–10 days post partum. Treatment was not associated with any untowards effects or discomfort, mice were sacrificed 24 h following treatment and mammary tissues were evaluated using histology and RT-qPCR analysis for gene expression in comperison with LPS-treated mice. Although IMM infusion of bMSC elicited inflammatory response in the mammary glands, major differences were observed in comparison with the LPS-treated glands. Massive recruitment of blood neutrophils into the alveolar milk space is the hallmark of acute mastitis and was clearly visible in the LPS-treated mice (Fig. 7A-B). Interestingly, in the bMSC-treated glands blood neutrophils were recruited into the parenchyma surrounding the alveoli and were not visible in the alvolar milk space (Fig. 7C-D). Furthermore, gene expression analysis also revealed major differences (Fig. 8), most strickingly, while the expression of Cxcl1 (Fig. 8; top middle panel) was increased, Cxcl2 (Fig. 8; top right panel) was in the normal levels 24 h after IMM bMSC treatment. This difference might explain the abrogated neutrophil recruitment observed in bMSC-treated glands.

Next, we evaluated the therapeutic effect of bMSC for LPS mastitis in lactating mice. Based on prieviously published data revealing the presence of early inflammatory changes in this model system [37], LPS-challenged glands were treated as above at 6 h after challenge. Mice were scarificed 24 h after LPS challenge and histological evaluation of LPS/bMSC-treated glands revealed massive recruitment of blood neutrophils into the alveoli and increased gene expression of all inflammatory markers comparable with non-treated LPS-challenged glands (Fig. 8). We concluded that local IMM bMSC treatment did not affect the outcome of IMM LPS challenge in this model system.

**Fig. 9:**
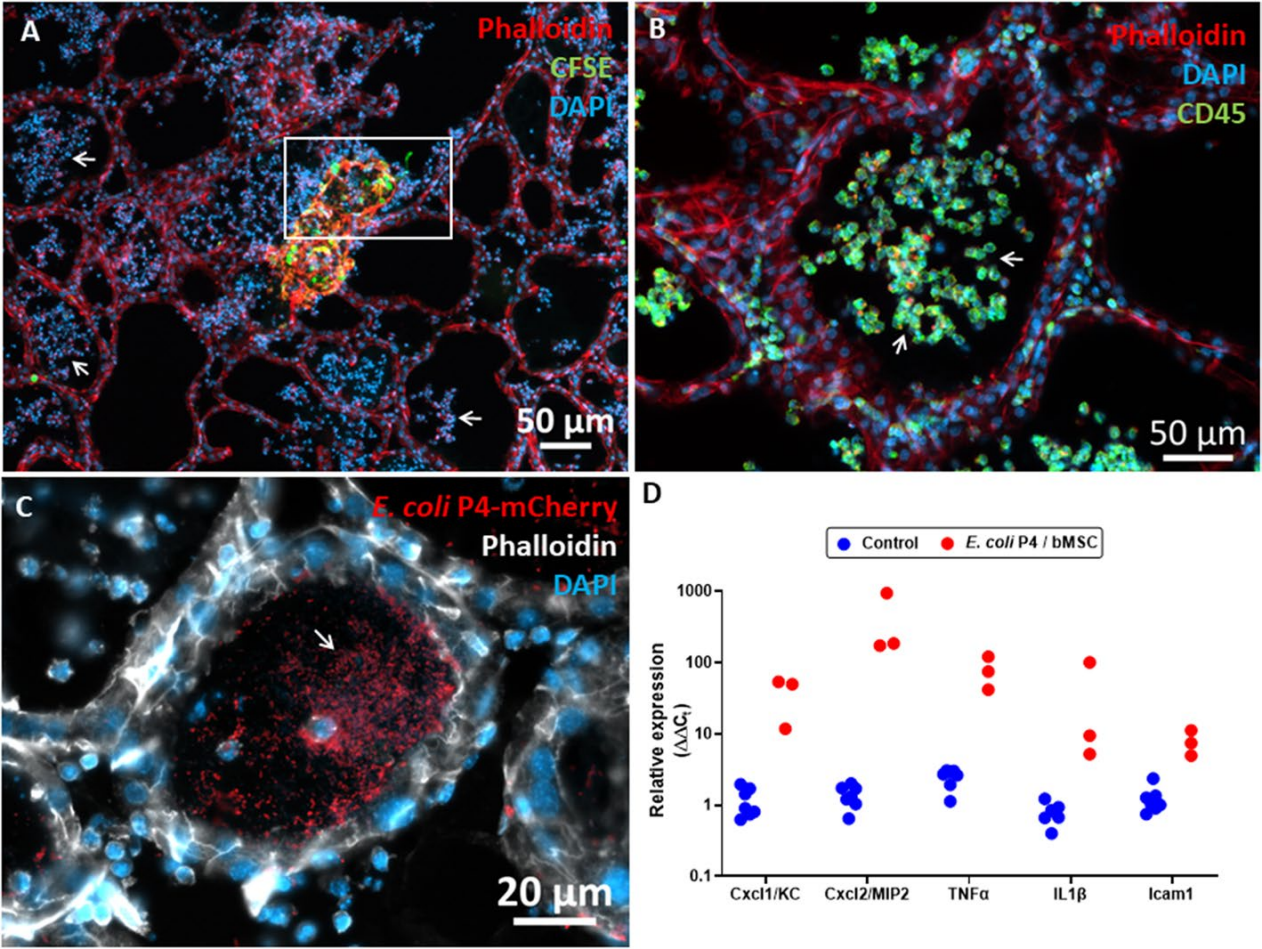
*E. coli* murine mastitis treated by intramammary (IMM) infusion of bMSC. Lactating BALB/c mice were challenged by IMM infusion of 10^4^ CFUs of *E. coli* P4-GFP bacteria and treated 6 h later by IMM infusion of 200,000 bMSC. Mammary tissues were harvested 24 h after challenge and analyzed using fluorescence microscopy (**A**-**C**) and gene expression using qPCR (**D**). Mastitis is characterized by massive infiltration of neutrophils into alveolar and tubular milk spaces (white arrows in **A**-**C**) and increased gene expression of inflammation markers (**D**). Representative images of fluorescence staining using DAPI (blue) phalloidin (red in **A**-**B**, white in **C**), and anti-murine CD45 antibodies (green in **B**). Fluorescing GFP bacteria interacting with neutrophils and epithelial cells are visible in C. CFSE-labelled bMSC are visible in the lumen of a large milk tubule (green in **A**) and boxed area is enlarged and better visible in Supplementary Figure S7. Using RT-qPCR the relative expression of Cxcl1 (KC), Cxcl2 (MIP2), TNFɑ, IL1β, and Icam1 genes was quantified relative to RNA samples extracted from the mammary tissues of lactating normal age-matched control mice (**D**). Scale bars 50 µm (**A**-**B**) and 20 µm (**C**)

The therapeutic failure of MSCs infused into the inflamed milk spaces might be attributed to inability of these cells to function and survive in this composite environment. Our next aim was to study the compatibility of MSCs with the inflamed mammary gland niche. To this end, we used fluorescence microscopy to visualize CFSE-labeled bMSCs infused via the teat canal 6 h after IMM challenge with *E. coli* strain P4 bacteria. This mammary pathogenic strain was selected because it is known to be milder, less virulent and non-cytotoxic but still able to colonize the murine mammary gland and to elicits mastitis. As expected, 24 h after challenge with the P4 strain mammary infection and inflammation was characterized by massive neutrophil recrutment into the alvelar milk spaces (Fig. 9A-C) and increased expression of the following genes; Cxcl1, Cxcl2, Tnf, Il1b, and Icam1 (Fig. 9D). Fluorescently-labled bMSCs infused 6 h after challenge were visible in some of the alveoli interacting with recruited blood neutrophils (Fig. 9A). Interestingly, bMSC in the alverolar milk space lost their normal mesenchymal motrphology and viability showing condensation of F-actin and loss of nuclei (Fig. 9A and Supplementary Figure S7).

**Fig. 10:**
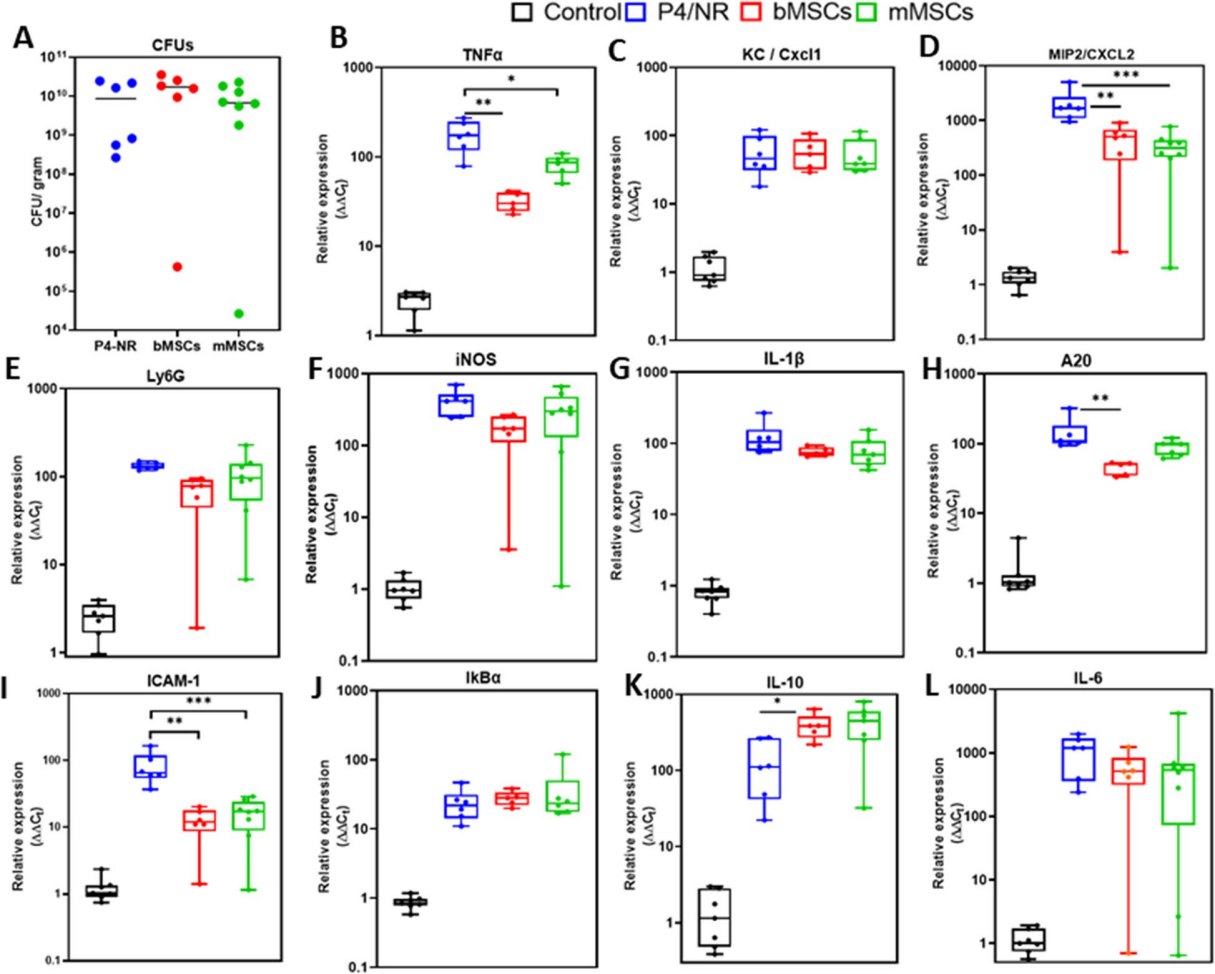
Effects of systemic bMSC and mMSC injectionon murine coliform mastitis. Lactating mammary glands were challenged with mammary pathogenic *E. coli* strain P4-NR bacteria by intramammary infusion. Mice were randomly allocated to the following groups; non-treated (blue), bMSC-treated (red) and mMSC-treated (green). MSC treatment was applied by intravenous infusion 6 h after challenge and all animals were sacrificed 24 h after challenge, mammary tissues were harvested for total bacterial counts and RNA extraction for RT-qPCR analysis. Total bacterial counts were not different between MSC-treated and non-treated animals (**A**). Box plots (**B**-**L**) presenting relative expression of inflammatory markers in mammary tissues of normal lactating animals (Control; black) and experimental groups. Each data point within box plots represents a single gland and each experimental group was compared to the normal control group or between groups by non-parametric Mann–Whitney two-independent-samples test using GraphPad Prism 6 (GraphPad Software, Inc.). *P* value of 0.05 or less was considered significant. Non-significant; (ns); *P* > 0.05, *P ≤ 0.05; **P ≤ 0.01, ***P ≤ 0.001, ****P ≤ 0.0001

Our results confirmed that the inflamed murine mammary gland is not compatible with MSC viability and survival. Henceforth, we next used the intravenous route for the application of systemic MSC treatment in murine mastitis. Lacating BALB/c mice were challenged with *E. coli* strain P4-NR by IMM infusion therough the teat canal. Bovine or mouse MSCs were injected intravenously 6 h after bacterial challenge and tissues were harveted 24 h after challenge. Either treatment did not affect the total bacterial counts in the glands (Fig. 10A), however, both treatments significantly affected the inflammatory response (Fig. 10B-L). Most notabley, the relative expression of TNFɑ (Fig. 10B), Cxcl2 (Fig. 10D), and Icam1 (Fig. 10I) was significantly lower following both bovine and murine MSCs intravenous treatment.

### Effects of MSC on normal cow mammary glands

The putative use of bMSCs as mastitis therapeutic requires prior evaluation of their effects on the normal lactating mammary glands in dairy cows. To this end, 6 normal quarters of 3 dairy cows were infused with 10^6^ CFSE-labeled bMSC suspended in normal saline.

**Fig. 11:**
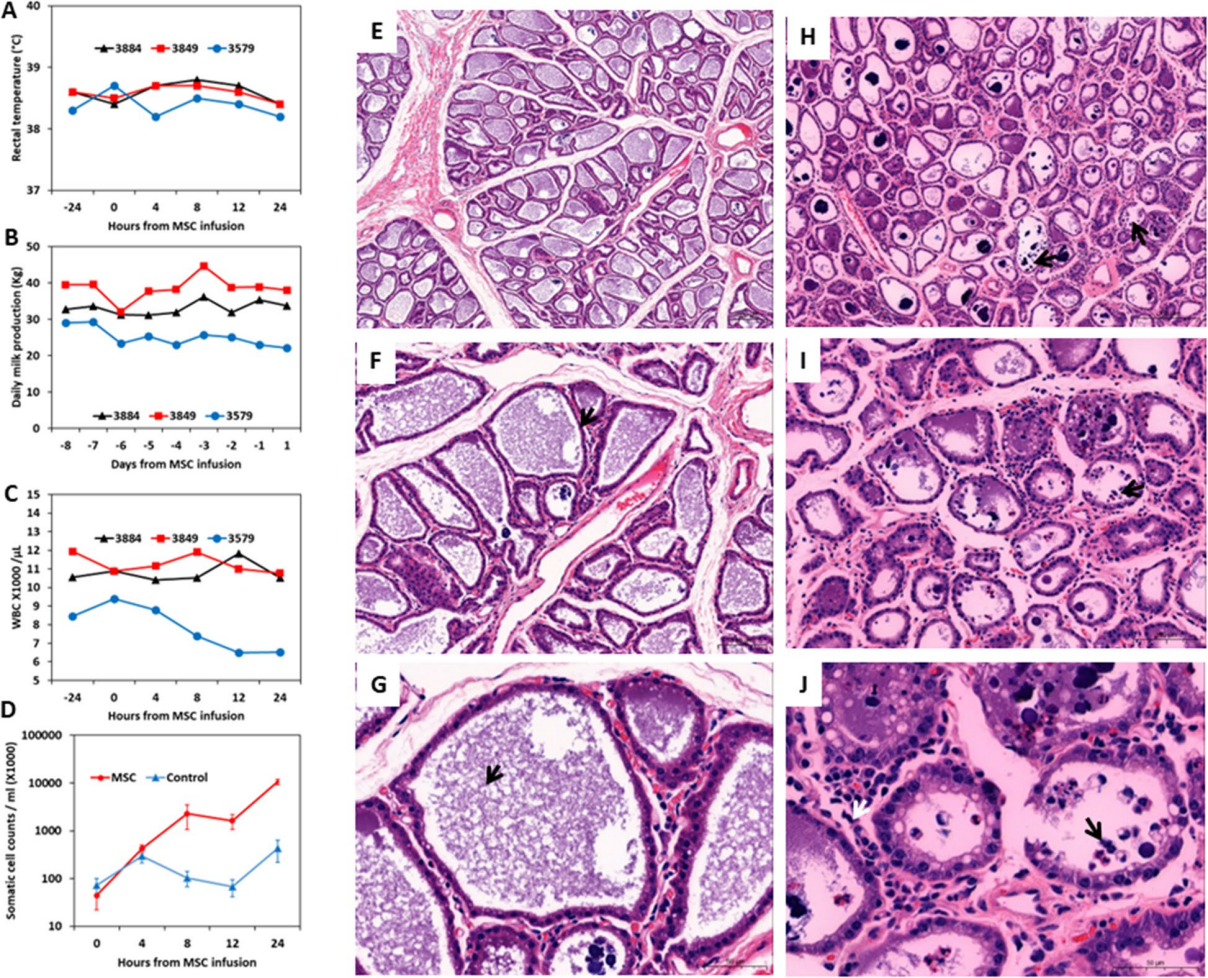
Intramammary infusion of bMSC in normal lactating dairy cows. Rectal temperature (**A**), milk production (**B**), total blood white cell counts (WCC in **C**), and SCC (**D**) were recorded prior to intramammary (IMM) bMSC treatment and 8 hourly thereafter. Representative microscopic images of formalin-fixed paraffin-embedded H&E stained tissue sections from control quarters (**E**–**G**) and bMSC-treated quarters (**H**-**J**). Quarters treated with bMSC demonstrated considerable elevation of SCC while control quarters showed only mild increase (**D**). Histopathological examination of bMSC treated quarters revealed sparse infiltration of neutrophils into the parenchyma (white arrow in **J**) and rare alveoli infiltrated with neutrophils (black arrows in **H**-**J**). Scale bars, 200 µm (**E**&**H**), 100 µm (**F**&**I**) and 50 µm (**G**&**J**)

Based on clinical examinations (Fig. 11A), milk production (Fig. 11B), blood biochemistry, complete and differential blood cell counts (Fig. 11C), SCC (Fig. 11D), CMS and milk culture results, all cows and quarters were normal and free of any signs of either systemic or mammary disease signs. Quarters treated with bMSC demonstrated considerable elevation of total SCC while control quarters showed only mild increase (Fig. 11D). Histopathological examination of bMSC-untreated quarters (Fig. 11E-G) compared to treated quarters revealed sparse infiltration of neutrophils into the parenchyma (white arrow in Fig. 11J) and rare alveoli infiltrated with neutrophils (black arrows in Fig. 11H-J). We could not demonstrate fluorescently labelled bMSC in the udder tissues or lymph nodes.

## Discussion

Our in-vitro co-culture studies applying MSC to mammary epithelial and uterine endometrial cells further support previous studies demonstrating their immunomodulatory and anti-inflammatory effects [10]. The barrier epithelial cells in the mammary gland and uterus are the first line of defense against invading bacterial pathogens ascending through the mammary teat canal or the cervix, respectively. In these cells, activation and signaling of inflammation by microbial cues are mediated through the activation of NF-kB, the master regulator of inflammation [36]. Furthermore, we also show that applying MSC to mammary epithelial cells or endometrial cells significantly diminished their inflammatory response, potentially supporting their therapeutic use for mastitis and metritis. However, significant discrepancies were previously reported between in-vitro and in-vivo data from animal models [38]. To this end, we established a novel murine metritis model system and adopted the *E. coli* mastitis model in lactating mice [18, 19, 24] to study the biology, feasibility, safety and efficacy of MSC therapy in these disease conditions.

Information regarding the pathophysiology of metritis and virulence mechanisms of utero-pathogenic organisms is scarce [39, 40]. The relevance of previously published animal model systems to the disease in dairy cows is doubtful due to the use of irrelevant challenge organisms and challenge routes. Since utero-pathogenic *E. coli* bacteria were implicated in bovine metritis [41], we have used a strain isolated from a field case of metritis in a dairy cow. Furthermore, bovine metritis is an ascending infection hence we have used the transcervical route for the installation of the challenge organisms. Lastly, since the estrus cycle profoundly affects structural and cellular elements and susceptibility to infection of the uterus, we have used sexually mature synchronized diestrus mice in the challenge studies.

Taken together, all the above resulted in a reliable and highly repeatable murine metritis model that was used to study the safety and therapeutic efficacy of MSC. We show here that transcervical application of MSC to the infected murine uterus results in reduced bacterial counts and increased inflammatory response (Ly6G, IL1β and metritis score) 24 h after challenge. Although these preliminary results are encouraging, considering the time scale of the disease in dairy cows, these effects should be further analyzed at later time points.

Interestingly, in the control groups, inflammatory response was elicited following transcervical infusion of similar volumes of PBS or MSCs suspended in PBS. While these results underscore the safety of transcervical MSC infusion, they might also demonstrate the utility of PBS or saline infusion of the uterus, which is commonly used by field veterinarians for the treatment of metritis [42, 43]. Previous studies demonstrated the ability of mechanical stretch of the myometrium to provoke proinflammatory chemokines and cytokines expression and release followed by recruitment of peripheral immune cells into the uterus [44, 45]. The luminal space of the diestrus virgin mouse is very small and the transcervical intrauterine infusion procedure employed in this project undoubtedly resulted in mechanical stretch of the myometrium. We can only speculate that stretch-induced inflammatory response, which is physiologically important in the pregnant uterus, is considerably different from inflammation elicited by luminal bacteria.

In this study, the observed effect of systemically or locally administered mMSC and bMSC on *E. coli* mastitis was less encouraging. The hallmarks of coliform mastitis, high bacterial counts, and neutrophil recruitment were not affected by MSC treatment. Nevertheless, systemic MSC treatment was associated with significantly reduced expression of TNFɑ, CXCL2, and ICAM-1 and increased expression of IL-10. These differences might indicate an immunomodulatory effect of systemic MSC on the inflamed mammary gland that could not be fully explored in this acute model system.

These systemic effects were observed, although we could not demonstrate MSC engraftment in the mammary gland, and this paradox of measurable effects devoid of evidence of cell engraftment was previously discussed [46]. Intravenous administration of MSCs is not associated with significant homing and engraftment in target tissues, as the vast majority of transplanted MSCs are entrapped in the pulmonary vasculature and subsequently cleared from the circulation [47]. Most probably, only a negligible percentage of MSCs administered systemically can reach the inflamed tissue. Instead, the biological activity of remotely engrafted MSCs might be mediated by the release of bioactive factors or MSC-derived extracellular vesicles such as apoptotic bodies, microvesicles and exosomes [6, 48, 49]. Further research is required to better understand these modes of action and molecular mechanisms, which might open novel prospective for cell-free MSC-based therapies.

## Supplementary Information

**Additional file 1: Supplementary Figure S1.** Bovine mesenchymal stromal cells (bMSC) form three dimensional structures when plated on Eph4 cells. **Supplementary Figure S2. **Massive recruitment of blood neutrophils into uterine lumen and endometrium following challenge with *E. coli* bacteria. **Supplementary Figure S3. **Recruitment of blood neutrophils in *E. coli *metritis. **Supplementary Figure S4.** Macrophages are not recruited into the uterine lumen in metritis.  **Supplementary Figure S5**. Increased expression of ICAM-1 in metritis. **Supplementary Figure S6**. Murine mesenchymal stromal cells (mMSC) transduced with the fluorescence reporter NFkB-Venus using lentivirus technology. **Supplementary Figure S7. **Bovine mesenchymal stromal cells (bMSC) in milk tubule following intramammary treatment of bacterial mastitis.

**Additional file 2: Supplementary Table 1.** Histopathological examination criteria for endometritis diagnosis. **Supplementary Table 2.** List of primers used for RT-qPCR analysis.

## Data Availability

All data generated or analysed during this study are included in this published article and its supplementary information files.
