# Supplementary material for: Mesenchymal stromal cells modulate infection and inflammation in the uterus and mammary gland
Source: BMC Vet Res. 2023 Mar 30;19:64. doi: 10.1186/s12917-023-03616-1 (PMC10061880; doi:10.1186/s12917-023-03616-1)
Supplement: Supplementary file 1 — Additional file 1: Supplementary Figure S1. Bovine mesenchymal stromal cells (bMSC) form three dimensional structures when plated on Eph4 cells. Supplementary Figure S2. Massive recruitment of blood neutrophils into uterine lumen and endometrium following challenge with E. coli bacteria. Supplementary Figure S3. Recruitment of blood neutrophils in E. coli metritis. Supplementary Figure S4. Macrophages are not recruited into the uterine lumen in metritis. Supplementary Figure S5. Increased expression of ICAM-1 in metritis. Supplementary Figure S6. Murine mesenchymal stromal cells (mMSC) transduced with the fluorescence reporter NFkB-Venus using lentivirus technology. Supplementary Figure S7. Bovine mesenchymal stromal cells (bMSC) in milk tubule following intramammary treatment of bacterial mastitis. [file 12917_2023_3616_MOESM1_ESM.pptx]

## Slide 1
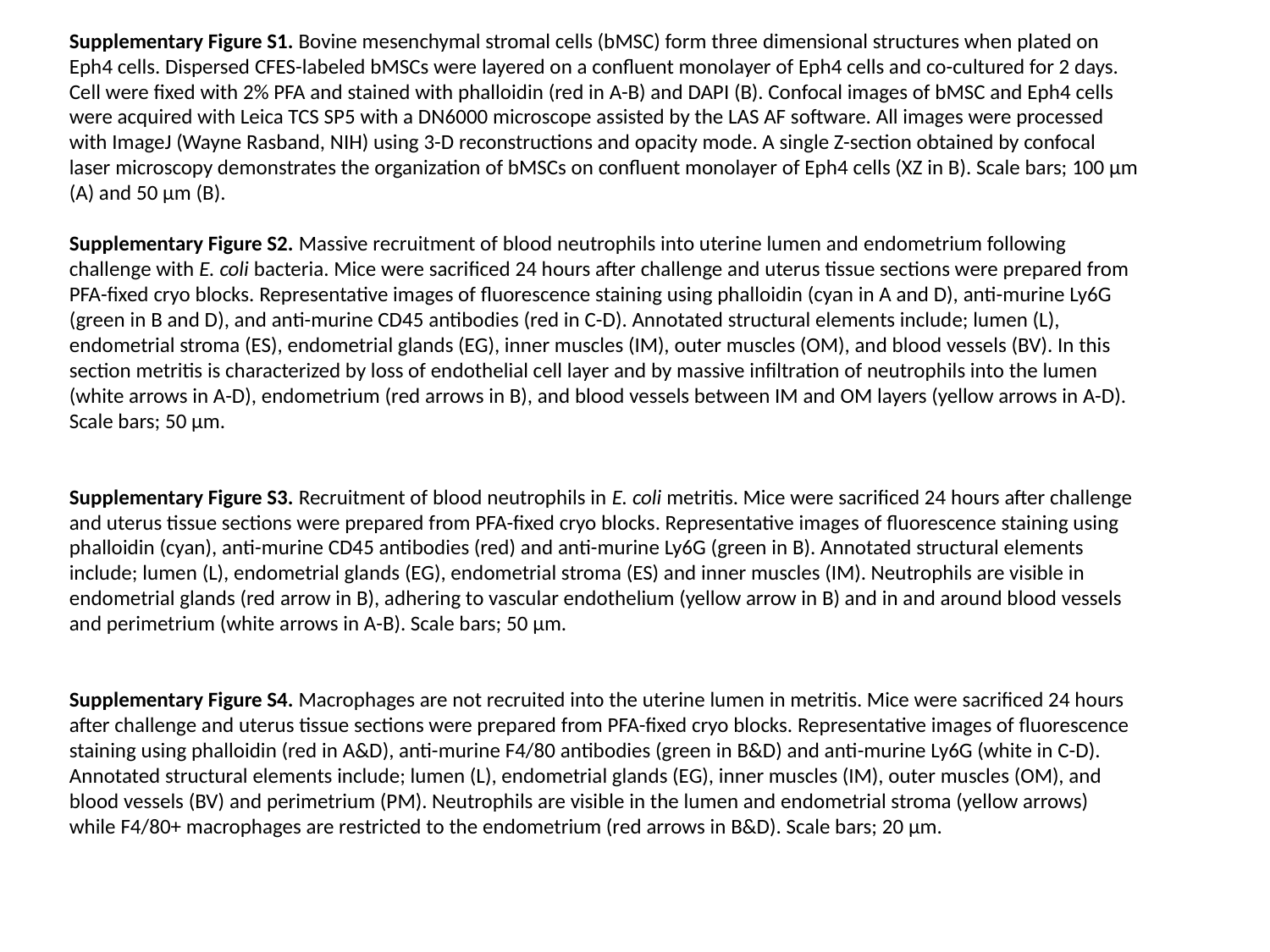

Supplementary Figure S1. Bovine mesenchymal stromal cells (bMSC) form three dimensional structures when plated on Eph4 cells. Dispersed CFES-labeled bMSCs were layered on a confluent monolayer of Eph4 cells and co-cultured for 2 days. Cell were fixed with 2% PFA and stained with phalloidin (red in A-B) and DAPI (B). Confocal images of bMSC and Eph4 cells were acquired with Leica TCS SP5 with a DN6000 microscope assisted by the LAS AF software. All images were processed with ImageJ (Wayne Rasband, NIH) using 3-D reconstructions and opacity mode. A single Z-section obtained by confocal laser microscopy demonstrates the organization of bMSCs on confluent monolayer of Eph4 cells (XZ in B). Scale bars; 100 µm (A) and 50 µm (B).
Supplementary Figure S2. Massive recruitment of blood neutrophils into uterine lumen and endometrium following challenge with E. coli bacteria. Mice were sacrificed 24 hours after challenge and uterus tissue sections were prepared from PFA-fixed cryo blocks. Representative images of fluorescence staining using phalloidin (cyan in A and D), anti-murine Ly6G (green in B and D), and anti-murine CD45 antibodies (red in C-D). Annotated structural elements include; lumen (L), endometrial stroma (ES), endometrial glands (EG), inner muscles (IM), outer muscles (OM), and blood vessels (BV). In this section metritis is characterized by loss of endothelial cell layer and by massive infiltration of neutrophils into the lumen (white arrows in A-D), endometrium (red arrows in B), and blood vessels between IM and OM layers (yellow arrows in A-D). Scale bars; 50 µm.
Supplementary Figure S3. Recruitment of blood neutrophils in E. coli metritis. Mice were sacrificed 24 hours after challenge and uterus tissue sections were prepared from PFA-fixed cryo blocks. Representative images of fluorescence staining using phalloidin (cyan), anti-murine CD45 antibodies (red) and anti-murine Ly6G (green in B). Annotated structural elements include; lumen (L), endometrial glands (EG), endometrial stroma (ES) and inner muscles (IM). Neutrophils are visible in endometrial glands (red arrow in B), adhering to vascular endothelium (yellow arrow in B) and in and around blood vessels and perimetrium (white arrows in A-B). Scale bars; 50 µm.
Supplementary Figure S4. Macrophages are not recruited into the uterine lumen in metritis. Mice were sacrificed 24 hours after challenge and uterus tissue sections were prepared from PFA-fixed cryo blocks. Representative images of fluorescence staining using phalloidin (red in A&D), anti-murine F4/80 antibodies (green in B&D) and anti-murine Ly6G (white in C-D). Annotated structural elements include; lumen (L), endometrial glands (EG), inner muscles (IM), outer muscles (OM), and blood vessels (BV) and perimetrium (PM). Neutrophils are visible in the lumen and endometrial stroma (yellow arrows) while F4/80+ macrophages are restricted to the endometrium (red arrows in B&D). Scale bars; 20 µm.

## Slide 2
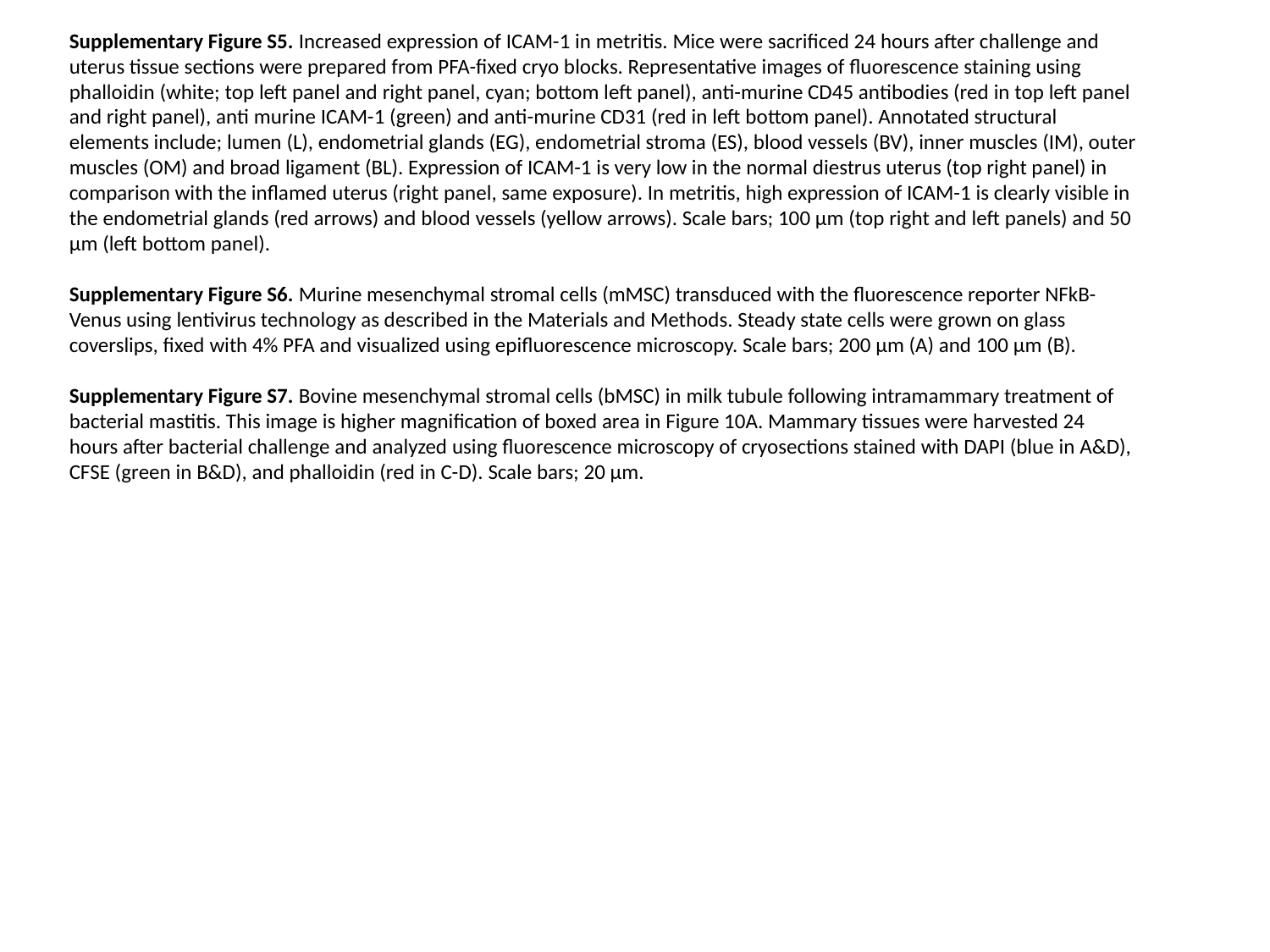

Supplementary Figure S5. Increased expression of ICAM-1 in metritis. Mice were sacrificed 24 hours after challenge and uterus tissue sections were prepared from PFA-fixed cryo blocks. Representative images of fluorescence staining using phalloidin (white; top left panel and right panel, cyan; bottom left panel), anti-murine CD45 antibodies (red in top left panel and right panel), anti murine ICAM-1 (green) and anti-murine CD31 (red in left bottom panel). Annotated structural elements include; lumen (L), endometrial glands (EG), endometrial stroma (ES), blood vessels (BV), inner muscles (IM), outer muscles (OM) and broad ligament (BL). Expression of ICAM-1 is very low in the normal diestrus uterus (top right panel) in comparison with the inflamed uterus (right panel, same exposure). In metritis, high expression of ICAM-1 is clearly visible in the endometrial glands (red arrows) and blood vessels (yellow arrows). Scale bars; 100 µm (top right and left panels) and 50 µm (left bottom panel).
Supplementary Figure S6. Murine mesenchymal stromal cells (mMSC) transduced with the fluorescence reporter NFkB-Venus using lentivirus technology as described in the Materials and Methods. Steady state cells were grown on glass coverslips, fixed with 4% PFA and visualized using epifluorescence microscopy. Scale bars; 200 µm (A) and 100 µm (B).
Supplementary Figure S7. Bovine mesenchymal stromal cells (bMSC) in milk tubule following intramammary treatment of bacterial mastitis. This image is higher magnification of boxed area in Figure 10A. Mammary tissues were harvested 24 hours after bacterial challenge and analyzed using fluorescence microscopy of cryosections stained with DAPI (blue in A&D), CFSE (green in B&D), and phalloidin (red in C-D). Scale bars; 20 µm.

## Slide 3
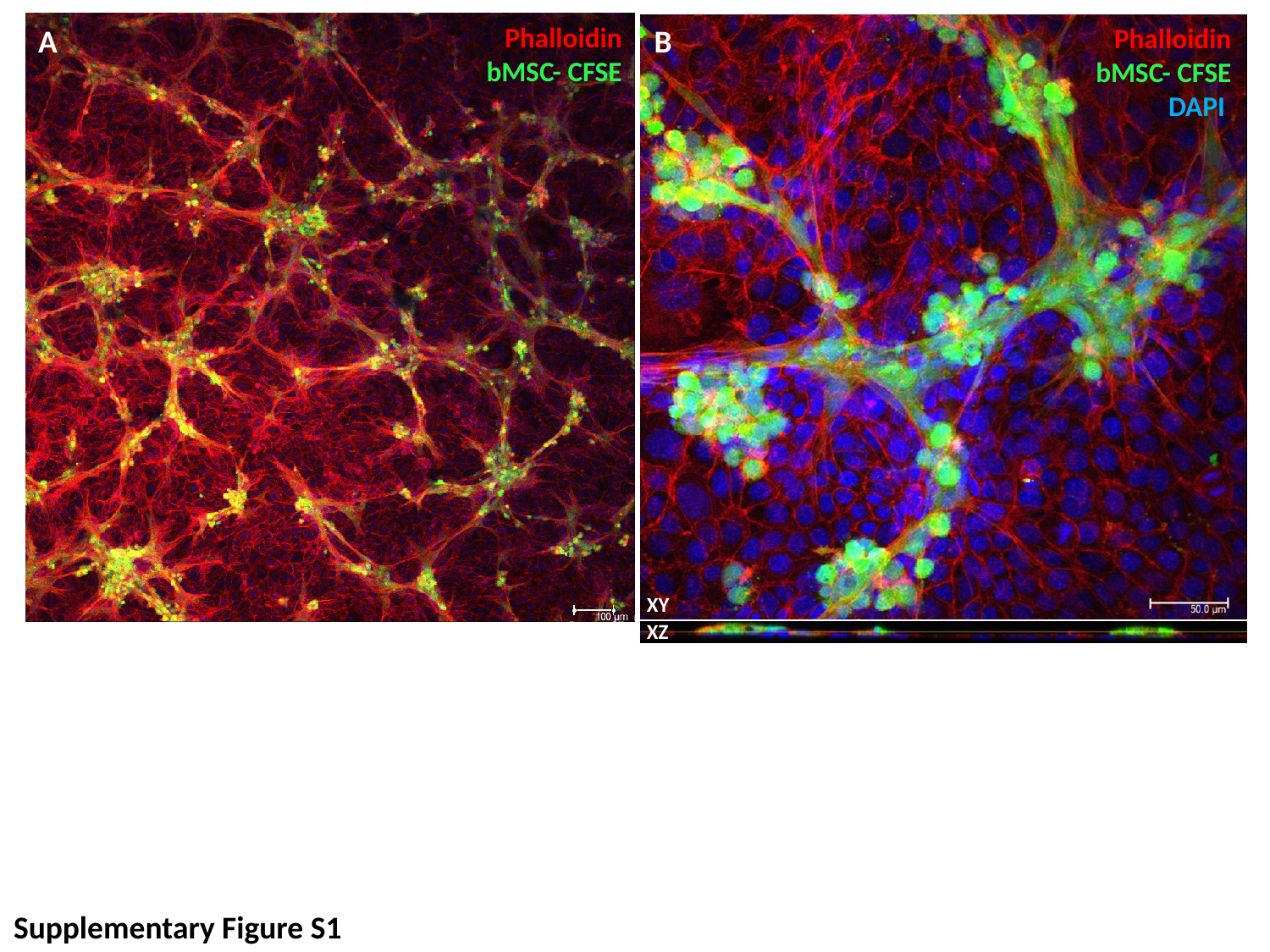

Phalloidin
bMSC- CFSE
A
B
Phalloidin
bMSC- CFSE
DAPI
XY
XZ
Supplementary Figure S1

## Slide 4
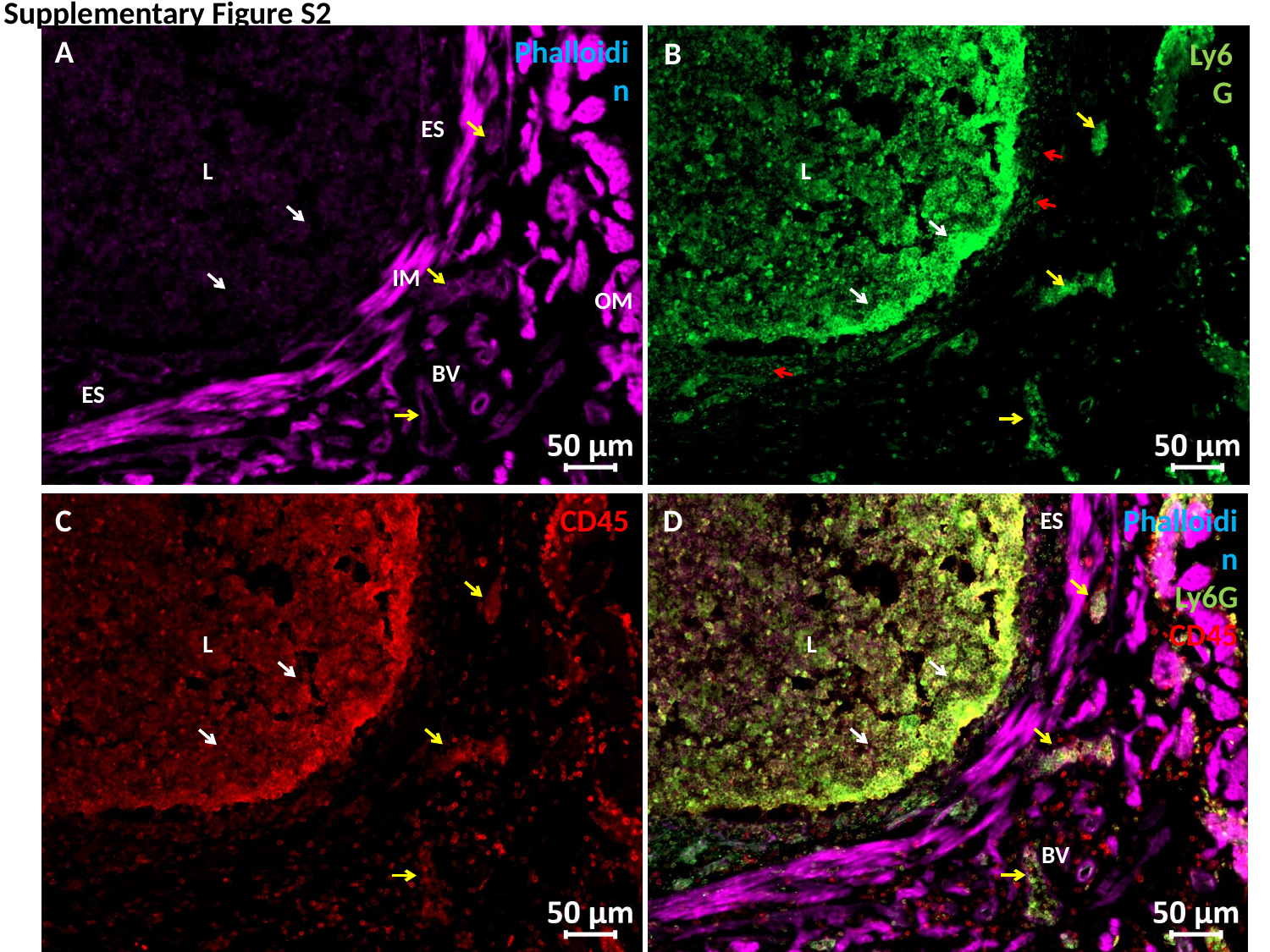

Supplementary Figure S2
A
Phalloidin
B
Ly6G
ES
L
L
IM
OM
BV
ES
D
Phalloidin
Ly6G
CD45
C
CD45
ES
L
L
BV

## Slide 5
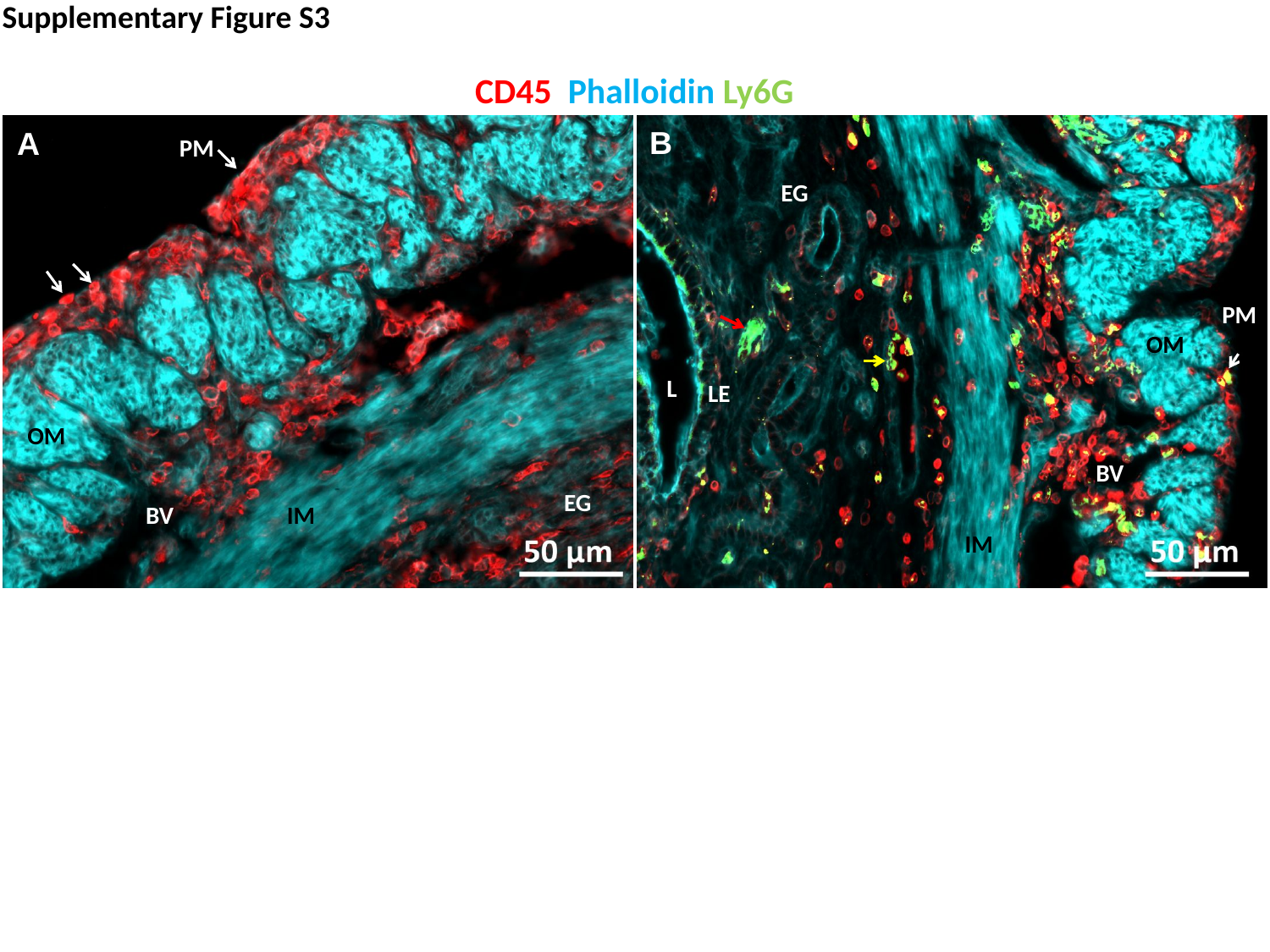

Supplementary Figure S3
CD45 Phalloidin Ly6G
B
A
PM
EG
PM
OM
L
LE
OM
BV
EG
BV
IM
IM

## Slide 6
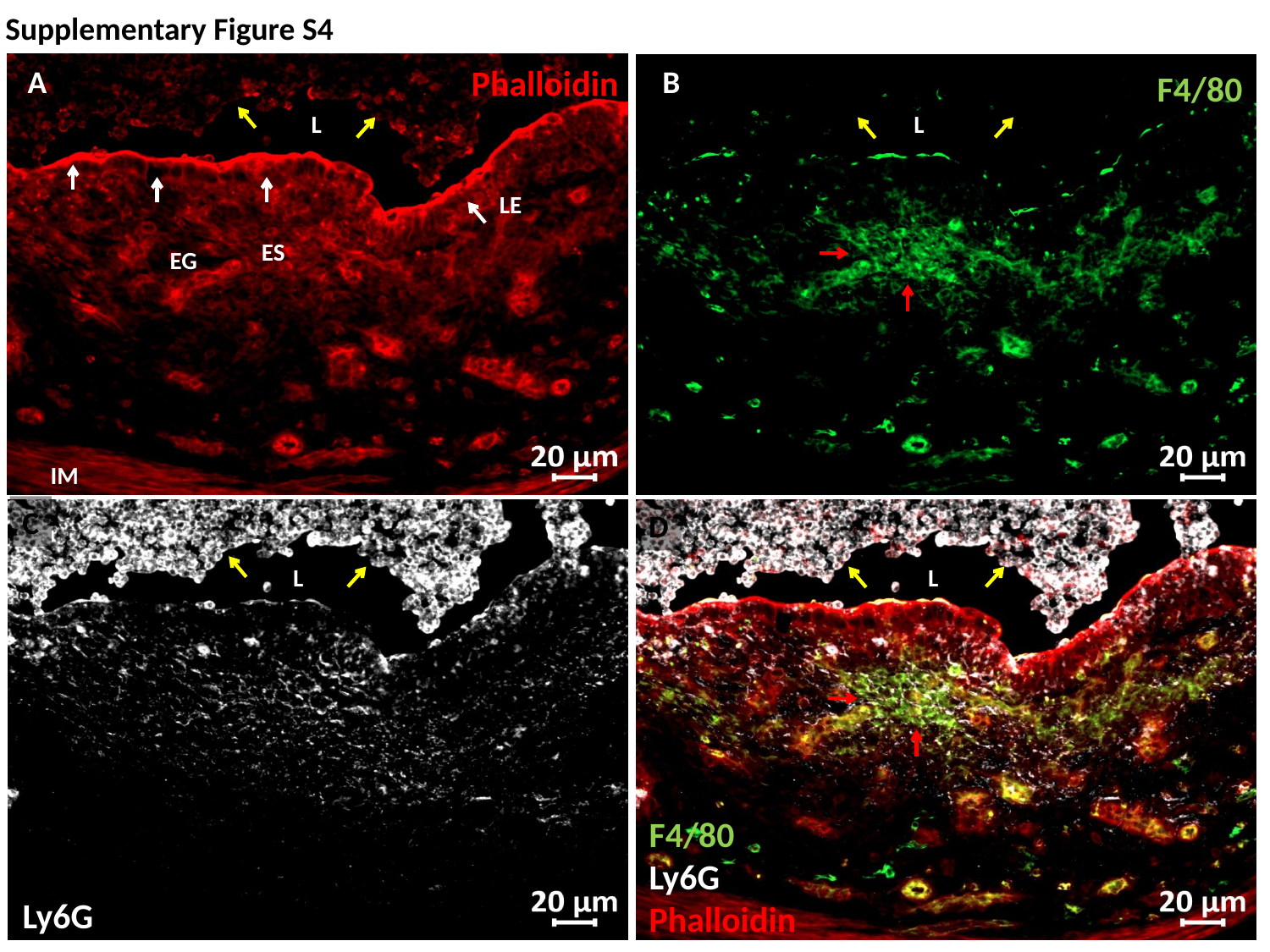

Supplementary Figure S4
Phalloidin
A
B
F4/80
L
L
LE
ES
EG
IM
C
D
L
L
F4/80
Ly6G
Phalloidin
Ly6G

## Slide 7
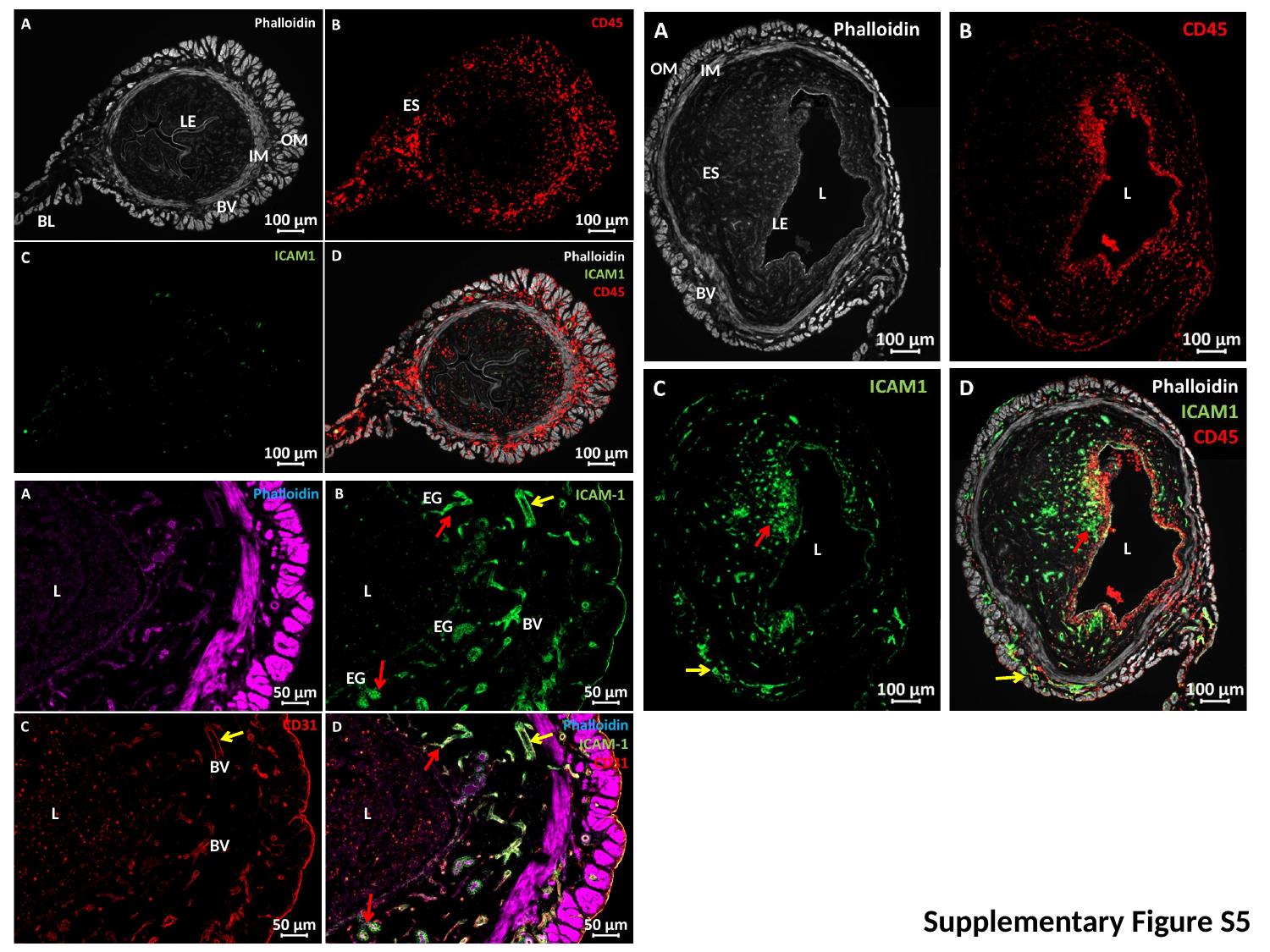

OM
IM
ES
LE
OM
IM
ES
L
L
BV
BL
LE
BV
EG
L
L
L
L
BV
EG
EG
BV
L
L
BV
Supplementary Figure S5

## Slide 8
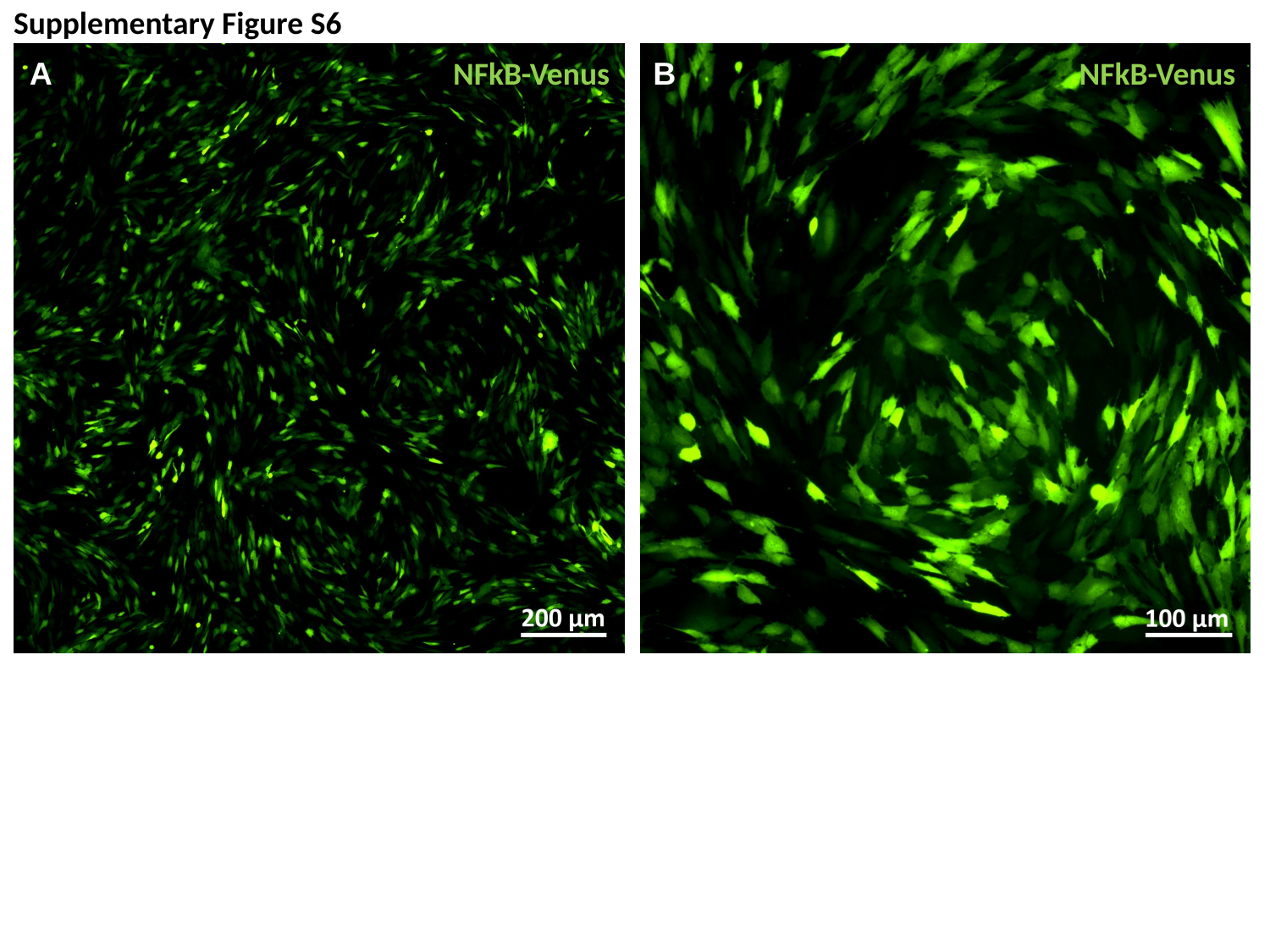

Supplementary Figure S6
A
NFkB-Venus
B
NFkB-Venus

## Slide 9
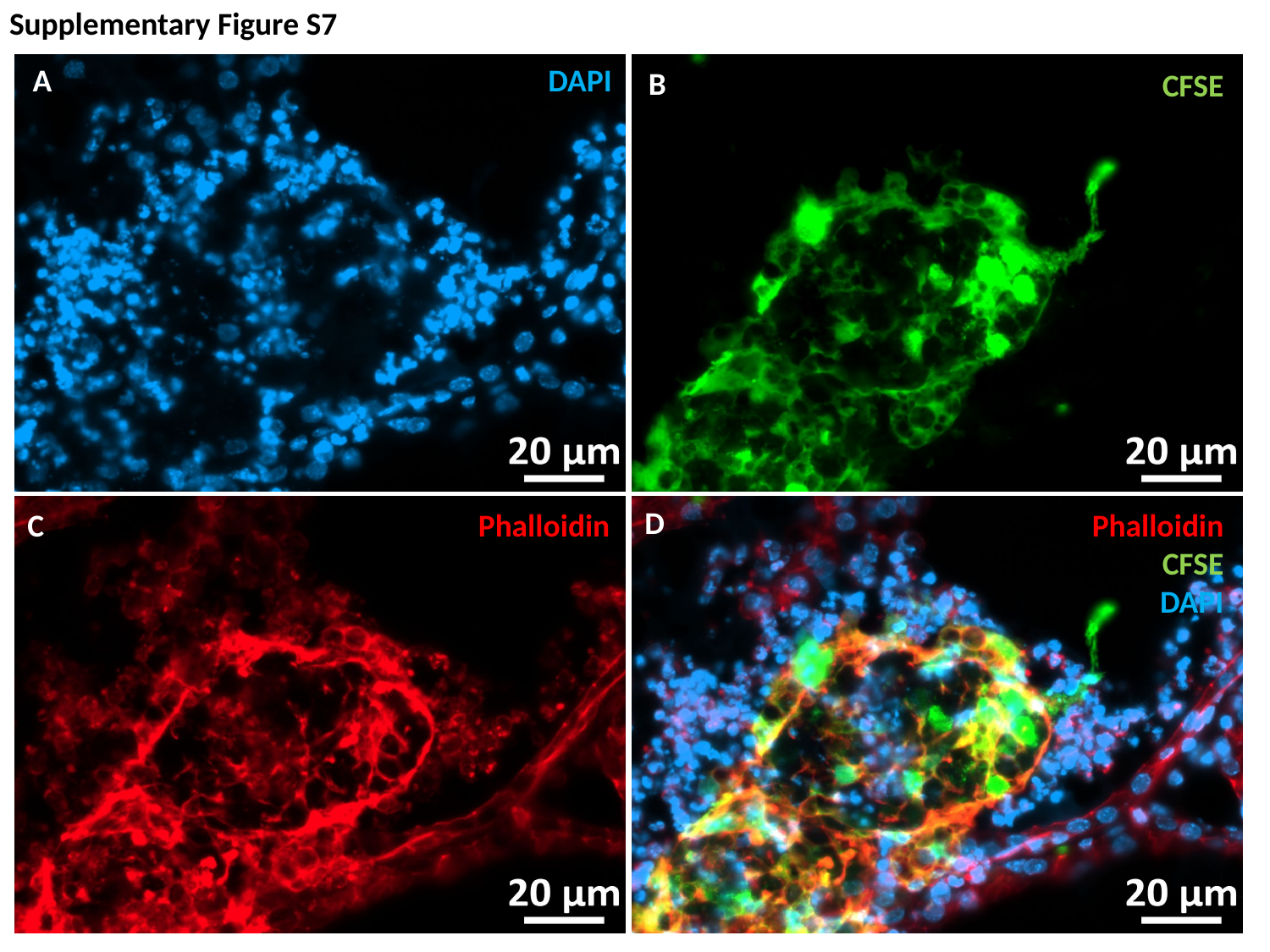

Supplementary Figure S7
A
DAPI
B
CFSE
D
C
Phalloidin
Phalloidin
CFSE
DAPI
