# Supplementary material for: Mesenchymal stromal cells modulate infection and inflammation in the uterus and mammary gland
Source: BMC Vet Res. 2023 Mar 30;19:64. doi: 10.1186/s12917-023-03616-1 (PMC10061880; doi:10.1186/s12917-023-03616-1)
Supplement: Supplementary file 2 — Additional file 2: Supplementary Table 1. Histopathological examination criteria for endometritis diagnosis. Supplementary Table 2. List of primers used for RT-qPCR analysis. [file 12917_2023_3616_MOESM2_ESM.docx]

**Supplementary Table 1.** Histopathological examination criteria for endometritis diagnosis.

| Variable | **Category** | Score (range) |
| --- | --- | --- |
| Epithelium |  | Score (0–10) |
| Height | Columnar | 0 |
|  | Cuboidal | 1 |
|  | Flattened | 2 |
| Epithelial damage | Absent | 0 |
|  | Mild | 1 |
|  | Moderate | 2 |
|  | Ulcer | 3 |
| Inflammatory cell type | Absent | 0 |
|  | Mononuclear | 1 |
|  | Polymorphonuclear | 2 |
| Infiltrate intensity | Absent | 0 |
|  | Mild (≤5 cells/hpf; ×401) | 1 |
|  | Moderate (≥6–10 cells/hpf; ×40) | 2 |
|  | Severe (>10 cells/hpf; ×40) | 3 |
| Lamina propria |  | Score (0–5) |
| Inflammatory cell type | Absent | 0 |
|  | Mononuclear | 1 |
|  | Polymorphonuclear | 2 |
| Infiltrate intensity | Normal (≤20 cells/hpf; ×40) | 0 |
|  | Mild (≥21–40 cells/hpf; ×40) | 1 |
|  | Moderate (≥41–70 cells/hpf; ×40) | 2 |
|  | Severe (>70 cells/hpf; ×40) | 3 |
| Endometrial gland |  | Score (0–3) |
| infiltration intensity | Absent | 0 |
|  | mild (≤3 cells total in glands/hpf; x40) | 1 |
|  | moderate (≥4-8 cells total in glands/hpf; x40) | 2 |
|  | severe (≥9 cells total in glands/hpf; x40) | 3 |
| Vascular |  | Score (0–3) |
| vascular congestion | Absent | 0 |
|  | mild (≤10 RBC /hpf; x40) | 1 |
|  | moderate (≥11-40 RBC /hpf; x40) | 2 |
|  | severe (≥41 RBC /hpf; x40) | 3 |
| perimetrium |  | Score (0–3) |
| infiltration intensity | Absent | 0 |
|  | mild (≤1-3 cells /hpf; x40) | 1 |
|  | moderate (≥4-8 cells /hpf; x40) | 2 |
|  | severe (≥9 cells /hpf; x40) | 3 |
| lumen |  | score (0-3) |
| infiltration intensity | Absent | 0 |
|  | mild (≤1-10 cells /hpf; x40) | 1 |
|  | moderate (≥11-50 cells /hpf; x40) | 2 |
|  | severe (≥51 cells /hpf; x40) | 3 |
| Total |  | Score (0–27) |

hpf; Per high power field at the stated magnification (×10 or ×40).

**Supplementary Table 2.** List of primers used for RT-qPCR analysis.

| **Gene** | **Forward** | **Reverse** |
| --- | --- | --- |
| KC | CGCTCGCTTCTCTGTGCA | ATTTTCTGAACCAAGGGAGCT |
| IL-6 | GATGCTACCAAACTGGATATAATC | GGTCCTTAGCCACTCCTTCTGTG |
| TNFa | CATCTTCTCAAAATTCGAGTGACAA | TGGGAGTAGACAAGGTACAACCC |
| Ly6G | TGCCCCTTCTCTGATGGATT | TGCTCTTGACTTTGCTTCTGTGA |
| A20 | AAACCAATGGTGATGGAAACTG | GTTGTCCCATTCGTCATTCC |
| IkBa | GTCTCCCTTCACCTGACCAA | CAGCAGCTCACGGAGGAC |
| INOS | TTGGAGTTCACCCAGTTGTG | ACCAGAGGCAGCACATCAA |
| MIP2 | TGCCTGAAGACCCTGCCAAGG | GTTAGCCTTGCCTTTGTTCAG |
| Icam1 | CTGAGCTCCGCTGCTACCT | AGGCCCAGGGATCACAAC |
| IL-1 | GGTCAAAGGTTTGGAAGCAG | TGTGAAATGCCACCTTTTGA |
| IL-10 | TGGCCTTGTAGACACCTTGG | AGCTGAAGACCCTCAGGATG |
| IFNg | GGCCATCAGCAACAACATAA | GTTGACCTCAAACTTGGCAATA |
| ACTB | ATGGAGGGGAATACAGCCC | TTCTTTGCAGCTCCTTCGTT |
| GAPDH | GAAGGGCTCATGACCACAGT | GGATGCAGGGATGATGTTCT |
| **BOVINE PRIMERS:** | | |
| CD29 | GCCAAGCAGATGAAAACAGA | AATGTTGAATTTGTGCACCATC |
| CD73 | CAATGGCACGATTACCTG | GACCTTCAACTGCTGGATA |
| CD90 | CGCCCTCCTGCTAACAGTC | GGCGTGGTGGTGGTATTCT |
| CD105 | TGGTCAGTAACGAGGTGGTCA | AGTGTGGGCTGAGGTAGAGG |
| CD34 | CCTGAAGCTAAATGAGACCT | AACTTTCTGTCCTGTTGGTC |
| CD45 | AGCCACCATGTGAGAACAAA | TCACATCCAGGAGGTTCACA |
| bPSMB2 | GATGCGAAATGGTTATGAACTG | AGGTTTCGGCGAGTGAAAT |
| bCHMP2A | ACCGTGAGCGACAGAAGC | TGGCCATTTTCTTGATGTCC |
